# DBDMH-Promoted Methylthiolation in DMSO: A Metal-Free Protocol to Methyl Sulfur Compounds with Multifunctional Groups

**DOI:** 10.3390/molecules28155635

**Published:** 2023-07-25

**Authors:** Yong-Jun Zhou, Yong-Gan Fang, Kai Yang, Jian-Yun Lin, Huan-Qing Li, Zu-Jia Chen, Zhao-Yang Wang

**Affiliations:** 1 School of Chemistry, South China Normal University, GDMPA Key Laboratory for Process Control and Quality Evaluation of Chiral Pharmaceuticals, Guangzhou Key Laboratory of Analytical Chemistry for Biomedicine, Key Laboratory of Theoretical Chemistry of Environment, Ministry of Education, Guangzhou 510006, China; 2020022483@m.scnu.edu.cn (Y.-J.Z.); 2020022476@m.scnu.edu.cn (Y.-G.F.); jianyunlin@outlook.com (J.-Y.L.); 2022022611@m.scnu.edu.cn (H.-Q.L.); chenzujia2022@163.com (Z.-J.C.); 2 College of Pharmacy, Gannan Medical University, Ganzhou 341000, China; 3 School of Mechanical and Automotive Engineering, South China University of Technology, Guangzhou 510640, China

**Keywords:** 2(5*H*)-furanone, thiolation, transition metal-free, dimethyl sulfoxide, C-S bond construction

## Abstract

Organic thioethers play an important role in the discovery of drugs and natural products. However, the green synthesis of organic sulfide compounds remains a challenging task. The convenient and efficient synthesis of 5-alkoxy-3-halo-4-methylthio-2(5*H*)-furanones from DMSO is performed via the mediation of 1,3-dibromo-5,5-dimethylhydantoin (DBDMH), affording a facile route for the sulfur-functionalization of 3,4-dihalo-2(5*H*)-furanones under transition metal-free conditions. This new approach has demonstrated the functionalization of non-aromatic C_sp2_-X-type halides with unique structures containing C-X, C-O, C=O and C=C bonds. Compared with traditional synthesis methods using transition metal catalysts with ligands, this reaction has many advantages, such as the lower temperature, the shorter reaction time, the wide substrate range and good functional group tolerance. Notably, DMSO plays multiple roles, and is simultaneously used as an odorless methylthiolating reagent and safe solvent.

## 1. Introduction

**Figure 1:**
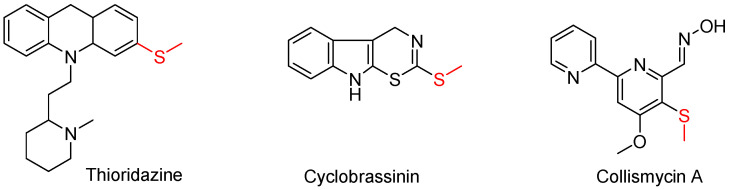
Some natural products and drugs containing the structure of the methyl sulfur group.

Sulfur is one of the most fundamental elements in the life system in the form of proteins and amino acids [1], and its rich valence states [2] are the chemical basis for its extensive use in medical drugs [3], pesticides [4] and organic luminescent materials [5,6]. In particular, their universal role in biological metabolism makes them crucial for organisms from the ocean to the terrestrial system [7]. Therefore, among the 362 sulfur-containing drugs listed by the US Food and Drug Administration (FDA), sulfide and its derivatives are one of a series of leading pharmaceutical active compounds [8]. For example, several nitro- and amino-bound sulfides are biologically active ingredients and are widely used to treat inflammation, Alzheimer’s disease, HIV, breast cancer, malaria and fungal-related diseases. In a word, among numerous sulfur-containing compounds, thioethers are an important structural component existing in many biological and pharmaceutical molecules [9] (some important examples with the structure of the methyl sulfur group and nitrogen-containing heterocyclic ring are shown in Figure 1 [10,11]).

In addition, thioethers are also important intermediates in organic synthesis, which can be transformed into sulfones [12] and sulfoxides [13], or used as the substrates in the Sonogashira reaction [14] and other reactions [15]. At present, the construction methods of methyl sulfur compounds are mainly the reduction of sulfoxides [16,17] and the reaction of aryl thiols with iodides [18] or dimethyl carbonate [19]. Among the methods mentioned above, some sources of sulfur have obvious defects. In particular, for the more commonly used reaction of thiols (thiophenols) [20,21], these sulfur reagents bring many shortcomings, such as unpleasant odors, toxicity and harsh reaction conditions. Therefore, the development and utilization of more stable, environmentally friendly and economical sulfur reagents for the synthesis of sulfide compounds still have important significance.

**Scheme 1:**
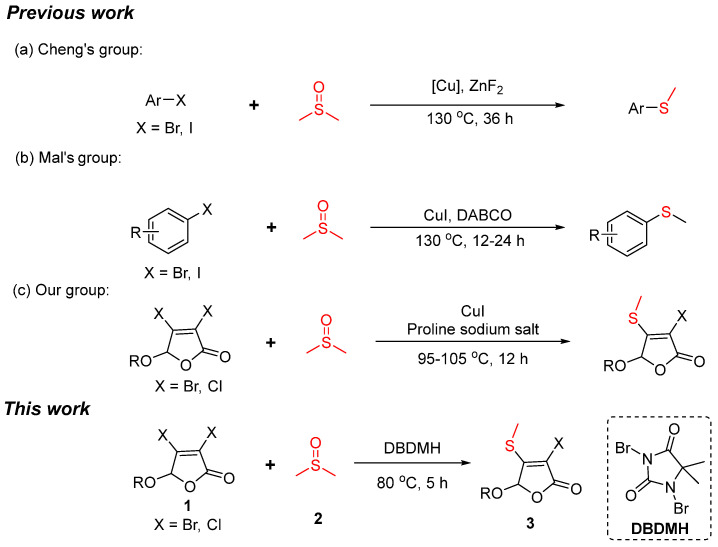
The synthesis of thioethers using DMSO.

Dimethyl sulfoxide (DMSO) is widely used as a high-quality solvent because of its high solubility for many organic and inorganic compounds [22,23]. In addition to being used as a solvent, DMSO has also been used as a multifunctional, inexpensive and safe reagent as a carbon [24,25], sulfur [26,27] and oxygen source [28,29] in many reactions. However, due to the low activity of reaction substrates (especially for chlorides) or the fact that these reactions often require a higher temperature and a longer reaction time, the synthesis research progress in using DMSO as a sulfur source with halogen-containing compounds to construct molecules with the methyl sulfur group is relatively slow. For example, in 2011, Cheng’s group [30] reported the copper-mediated methylthiolation of aryl halide and DMSO (Scheme 1a). Later, Mal’s group [31] further optimized the Cu-mediated method via the methylthiolation of halogenated aromatic hydrocarbons and DMSO by changing the reaction conditions (Scheme 1b).

In recent years, our group reported the methylthiolation reaction of the non-aromatic C_SP2_-X compound 3,4-dihalo-2(5*H*)-furanone with DMSO, further expanding the construction scope of methyl sulfur compounds when using halides and DMSO as substrates (Scheme 1c) [32]. Even so, it is a pity that, in these methods mentioned above, there are still shortcomings, such as the higher reaction temperature, the longer reaction time and the use of transition metal catalysis and ligands. Therefore, the green synthesis methods for preparing methyl sulfur compounds from readily available starting materials still need to be explored.

**Scheme 2:**
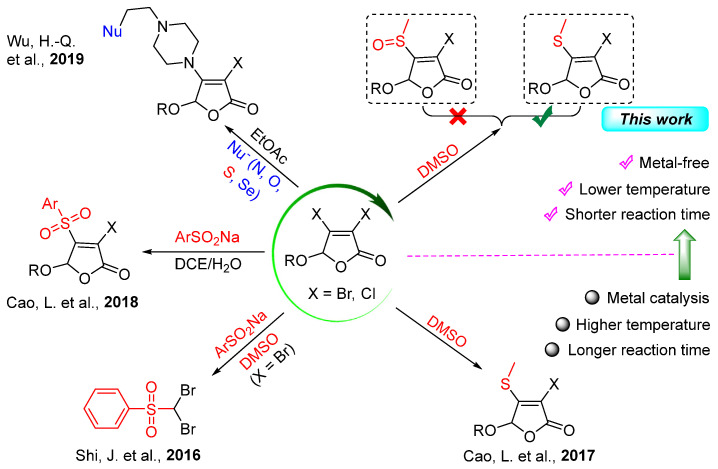
Green synthesis from 3,4-dihalo-2(5*H*)-furanone and various sulfur-containing reagents [32,40,41,43].

Sulfoxides are also a class of extremely important compounds because they are not only widely used as intermediates in synthesis but also as chiral ligands in various asymmetric catalysis [33,34]. In addition, sulfoxides can also be applied in many fields such as fine chemicals, pharmaceuticals, pesticides and functional materials [35,36]. On the basis of our interest in the synthesis of sulfur-containing compounds [37,38,39], especially the construction of a series of sulfur-containing compounds through the reaction of 3,4-dihalo-2(5*H*)-furanone with various sulfur-containing reagents (Scheme 2) [40,41,42,43], we aimed to introduce the sulfoxide group at the 4-position of 3,4-dihalo-2(5*H*)-furanone instead of the 4-halo group by controlling reaction conditions, in order to make the whole work of 2(5*H*)-furanone chemistry more systematical. However, as an unexpected result, a thioether structure instead of the sulfoxide group was found (Scheme 2).

It is worth noting that different 2(5*H*)-furanone compounds have been developed in many fields such as modern organic synthesis [44], drug molecular design [45] and natural product total synthesis [46] due to their potential high biological activities, such as antiviral [47] or anti-HIV activity [48]. In particular, the thioetherified 2(5*H*)-furanone can be a kind of potential drug molecule with good anticancer activity, as Li’s group reported before [49]. Therefore, it is quite important to develop a new method for the more efficient synthesis of the methylthioetherified 3,4-dihalo-2(5*H*)-furanone **3** (Scheme 1, this work). In addition, as a kind of compound containing multiple functional groups, the structure can be further modified in the next development and the obtained potential bioactive molecules may play an important role in drug development and design.

Thus, herein, on the basis of our group’s previous research on various functionalization reactions of 3,4-dihalo-2(5*H*)-furanones for bioactive compounds [50,51], we developed a simple, mild and transition metal-free method to realize the greener methylthiolation of 3,4-dihalo-2(5*H*)-furanones by further optimizing the conditions of the methylthiolation reaction from 3,4-dihalo-2(5*H*)-furanone and DMSO (Scheme 2, this work).

## 2. Results and Discussion

### 2.1. Optimization of Reaction Conditions

At the beginning of this study, 5-methoxy-3,4-dibromo-2(5*H*)-furanone **1a** was used as a model substrate to screen the reaction conditions in DMSO (Table 1).

Firstly, we optimized the activating agent (entries 1–3). Obviously, compared with *N*-bromosuccinimide (NBS) and *N*-chlorosuccinimide (NCS), when using 1,3-dibromo-5,5-dimethylhydantoin (DBDMH) as an activator, the obtained effect is the best, and the corresponding yield is as high as 87% (entry 3).

Next, based on the above results, we chose DBDMH as the activator to further optimize the dosage of DBDMH (entries 3–6). It can be found that, when its dosage is 1.5 equivalents, the conversion rate of this reaction is up to 93% (entry 5). Therefore, we selected 1.5 equiv. DBDMH to further optimize the temperature required for the reaction (entry 5 vs. entries 7–8). As can be seen from a series of temperature exploration experiments, 80 °C is more conducive to the reaction (entry 5).

Finally, we also explored the time required for the reaction (entry 5 vs. entries 9–10). It is obvious that prolonging or reducing the reaction time is not beneficial for improving the yield, and the best time is still 5 h (entry 5).

Thus, taking the reaction of 5-methoxy-3,4-dibromo-2(5*H*)-furanone **1a** in DMSO **2** as an example, the relatively ideal reaction conditions are as follows: using 0.4 mmol 5-methoxy-3,4-dibromo-2(5*H*)-furanone **1a** to react with 2 mL DMSO at 80 °C for 5 h, the isolated yield of product **3a** can reach 93%.

### 2.2. Investigation into the Range of Reaction Substrates

With the optimal conditions in hand, the substrate scope of this transformation was assessed. The results have been summarized, and are shown in Table 2.

Firstly, the tolerance of 3,4-dihalo-2(5*H*)-furanone substrates with different substituents R^1^ at the 5-position is examined when the halogen on the furanone ring is the bromine atom. Although the substituent groups at the 5-position on the furanone ring are different, the reaction can proceed smoothly (**3a**–**3j**, 74–93%). In particular, the reaction is well tolerated even for the substitution of a strong electron-withdrawing group of biphenyl, giving a 46% yield of product **3k**. 

In addition, as expected, the yield decreased slightly with the extension of the carbon chain and the enhanced steric hindrance effect (e.g., **3a** vs. **3d** vs. **3h**, 93% vs. 88% vs. 78%). Even so, it is satisfied that for the menthoxy group with large steric hindrance, the corresponding target product **3j** can be obtained with an isolated yield of 74%.

When the halogen is chlorine on the furanone ring, due to the activity difference between the bromine and chlorine atoms, the yield of the corresponding target products **3l**–**3t** is between 49 and 72%, and the steric hindrance effect of the substitution group at the 5-position is similar.

It is noteworthy that although the yield of the electron-withdrawing group was relatively low (e.g., Ph- in **3t**, 49%) compared with the yield data of the known compounds reported before [32], most of them were improved, especially for the cases where the previous yield was relatively low.

### 2.3. Structural Characterization Analysis

Firstly, the compounds synthesized herein were characterized using nuclear magnetic resonance (NMR) technology. From the ^1^H NMR spectra of the target compounds (please see the Supplementary Materials), it can be seen that the ^1^H NMR data of compounds **3a**–**3t** are consistent with the corresponding hydrogens in these target products. Similarly, the ^13^C NMR test results are also consistent.

**Figure 2:**
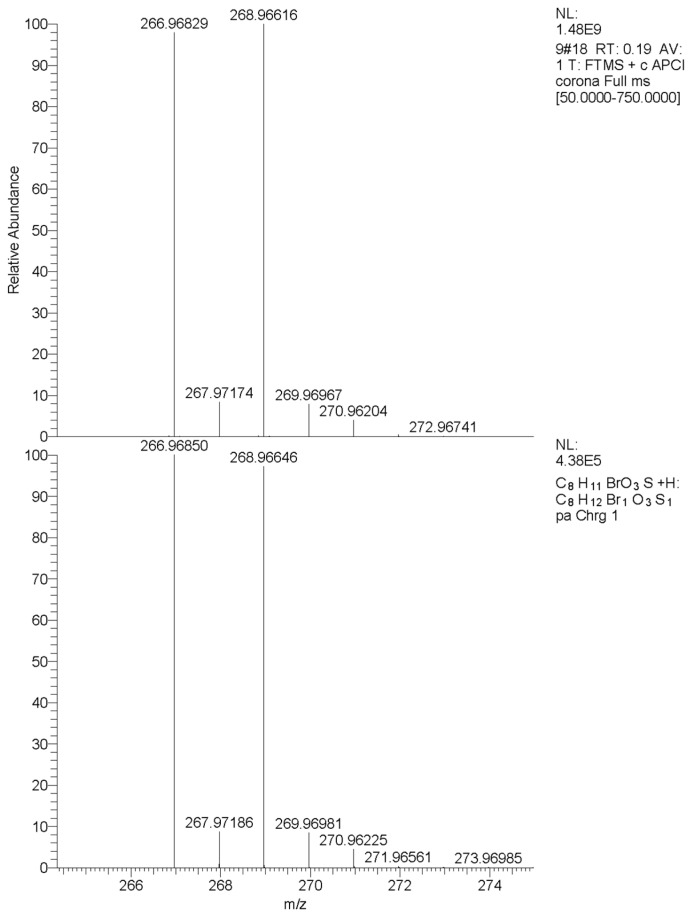
The HR-MS spectrum of compound **3c**.

In particular, for the synthesized new compounds, their spectra of high-resolution mass spectrometry (HR-MS) were also tested. Taking the target compound **3c** as an example, it can be found that HR-MS can also correspond well with the structure of compound **3c** (Figure 2). In short, from the analysis of the obtained HR-MS results in combination with other results, it is confirmed that DMSO can indeed react smoothly with 3,4-dihalo-2(5*H*)-furanones that are substituted with different alkoxy groups at the 5-position, giving the anticipated products.

Importantly, although nuclear NMR and mass spectrometry tests have confirmed the expected structure, in order to further determine the product structure, the single-crystal structure of **3a** (CCDC 2270564) [52] is also obtained (the detailed data can be seen in Table 3, and the corresponding molecular structure of **3a** can be seen in Table 2), which fully proves the structure of the anticipated product. Thus, the structures of the serial compounds are well characterized.

### 2.4. Mechanism Investigation and Gram-Scale Experiment

**Scheme 3:**
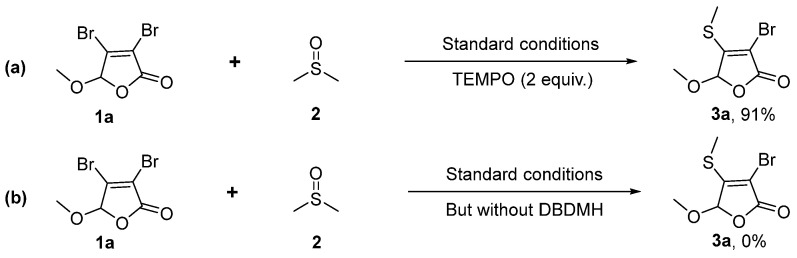
Control experiments.

To have a deeper understanding into the reaction process, we performed two control experiments accordingly. Firstly, we added two equiv. radical scavenger 2,2,6,6-tetra- methylpiperidin-1-yloxyl (TEMPO) to the reaction system. It was found that the corresponding compound **3a** can also be obtained in a 91% yield (Scheme 3a). And, compared with the reaction situation under the standard conditions, it is clear that the yield has almost no effect. This fully demonstrates that the reaction may not be involved in the pathway of radical participation.

Subsequently, the reaction under standard conditions for 5 h but without the addition of the activator DBDMH could not proceed smoothly (Scheme 3b). This indicates that DBDMH may be crucial in this transformation.

Compared with other brominating agents, such as NBS, or *N*-bromoacetamide, DBDMH as a special brominating agent has many advantages, e.g., high active bromine content, good storage stability and economic use [53,54,55]. In addition, acting as an oxidant in chemical synthesis, it also can be widely used in various transformations [56,57].

**Scheme 4:**
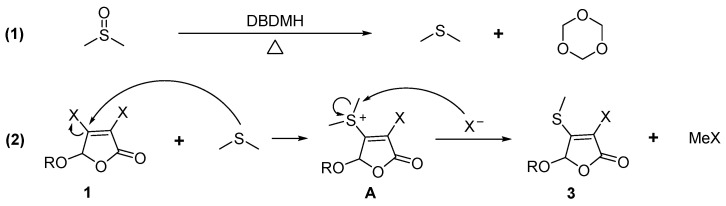
A plausible reaction pathway.

Thus, based on these relevant properties of DBDMH and the above-mentioned control experimental results, we proposed a possible reaction mechanism (Scheme 4), referring to the relevant literature reported before [58,59]:

Initially, with the promotion of DBDMH and heating, DMSO is successfully decomposed into dimethyl sulfide [60]. Subsequently, 5-alkoxy-3,4-dihalo-2(5*H*)-furanone **1** is attacked by dimethyl sulfide to form the intermediate **A**. Next, after a nucleophilic attack to intermediate **A** by halides in the reaction system, the target product **3** is obtained.

**Scheme 5:**
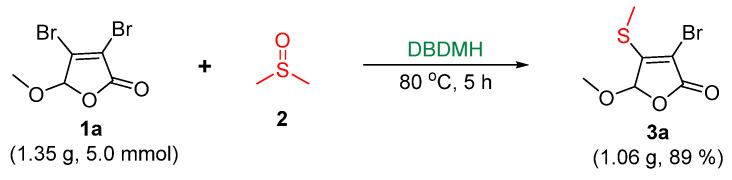
Gram-scale reaction.

In order to demonstrate the feasibility of synthetic applications of this transformation, a gram-scale experiment was carried out, and the reaction was performed with 5 mmol dosage. As shown in Scheme 5, when using 1.35 g **1a** to react with DMSO under standard conditions, the reaction can still be efficiently carried out, giving 1.06 g of the target compound **3a** with an excellent yield (89%).

Therefore, the experimental results show that the novel method of this interesting halide **1** and DMSO **2** under the simple and mild reaction conditions without the participation of transition metal is indeed successful, which is very important in the actual production for the drug development from potential bioactive compounds.

## 3. Materials and Methods

### 3.1. General Information

The spectra of ^1^H and ^13^C NMR were collected using an AVANCE NEO-600 in CDCl_3_, using tetramethylsilane (TMS) as an internal standard. High-resolution mass spectra (HR-MS) were obtained using a MAT 95XP mass spectrometer. Single-crystal X-ray analysis was obtained using Agilent Gemini E. Reactions were monitored using thin-layer chromatography (TLC) and visualized via UV light at 254 nm.

All reagents and solvents were purchased from the commercial sources and used without further purification.

### 3.2. Experimental Procedure for Intermediate Compounds ***1***

**Scheme 6:**
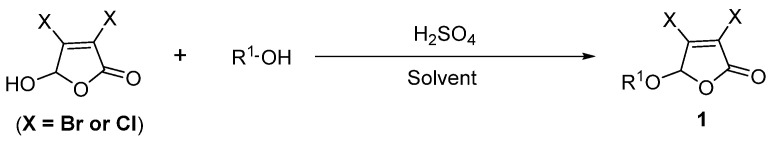
Synthesis route of intermediate **1**.

Different intermediate 5-alkoxy(aryloxy)-3,4-dihalo-2(*5H*)-furanones **1** were synthesized according to the procedure in the literature [43]. As shown in Scheme 6, after the slow addition of 1–2 drops of concentrated H_2_SO_4_ into the mixture of mucobromic acid or mucochloric acid (20 mmol) and the corresponding alcohol (30 mL) in a three-neck flask, the obtained mixture was heated to continue refluxing for 36–72 h.

Once the reaction was completed, the reaction mixture was quenched via the saturated solution of sodium chloride and extracted with ethyl acetate. Then, the organic layer was dried over anhydrous sodium sulfate solid. Finally, after filtration, the evaporation of the solvents under reduced pressure gave the crude product, which was further purified via column chromatography on silica gel to obtain intermediate **1**.

### 3.3. Experimental Procedure for Compounds ***3a***–***3t***

**Scheme 7:**
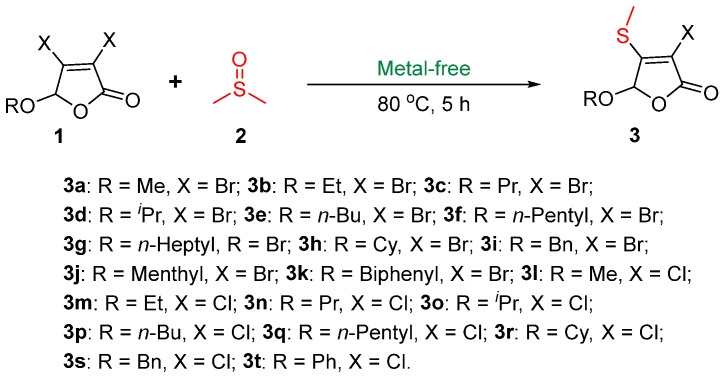
Synthesis route of target products **3a**–**3t**.

As shown in Scheme 7, 3,4-dihalo-2(5*H*)-furanone compound **1** (0.40 mmol) and DBDMH (0.60 mmol) were mixed in DMSO (2 mL), and the mixture was stirred at 80 °C for 5 h.

After the completion of the reaction, the reaction mixture was quenched with the saturated solution of sodium chloride (15 mL) and extracted with ethyl acetate (3 × 15 mL). Then, the organic layer was dried over anhydrous sodium sulfate solid. Finally, after filtration, the evaporation of the solvents under reduced pressure gave the crude product, which was further purified via column chromatography on silica gel to afford the desired product **3**.

### 3.4. Structural Characterization Data of Compounds ***3a***–***3t***

The structures of the serial compounds **3a**–**3t** were systematically characterized via NMR, HR-MS, etc., and the corresponding data are summarized in the following.

(1) 3-Bromo-5-methoxy-4-methylthiofuran-2(5*H*)-one (**3a**), yellow solid, m.p.: 84.7–85.9 °C (86.7–87.8 °C [32]), 89 mg, 93%; ^1^H NMR (600 MHz, CDCl_3_), *δ*, ppm: 2.60 (*s*, 3H, SCH_3_), 3.54 (*s*, 3H, OCH_3_), 5.90 (*s*, 1H, CH); ^13^C NMR (150 MHz, CDCl_3_), *δ*, ppm: 13.2, 54.8, 101.9, 105.6, 160.0, 164.9; ESI-HRMS, *m*/*z*: calcd for C_6_H_8_BrO_3_S [M + H]^+^: 238.9372, found: 238.9369.

(2) 3-Bromo-5-ethoxy-4-methylthiofuran-2(5*H*)-one (**3b**), yellowish oil, 85 mg, 84% (83% [32]); ^1^H NMR (600 MHz, CDCl_3_), *δ*, ppm: 1.31 (*t*, *J* = 7.2 Hz, 3H, CH_3_), 2.61 (*s*, 3H, SCH_3_), 3.73–3.92 (*m*, 2H, OCH_2_), 5.94 (*s*, 1H, CH); ^13^C NMR (150 MHz, CDCl_3_), *δ*, ppm: 13.2, 15.0, 64.6, 101.3, 105.4, 160.3, 165.0; ESI-HRMS, *m*/*z*: calcd for C_7_H_10_BrO_3_S [M + H]^+^: 252.9529, found: 252.9524.

(3) 3-Bromo-4-methylthio-5-propoxyfuran-2(5*H*)-one (**3c**), yellowish oil, 92 mg, 86%; ^1^H NMR (600 MHz, CDCl_3_), *δ*, ppm: 0.98 (*t*, *J* = 6.0 Hz, 3H, CH_3_), 1.67–1.74 (*m*, 2H, CH_2_), 2.61 (*s*, 3H, SCH_3_), 3.61–3.81 (*m*, 2H, OCH_2_), 5.95 (*s*, 1H, CH); ^13^C NMR (150 MHz, CDCl_3_), *δ*, ppm: 10.5, 13.2, 22.7, 70.4, 101.4, 105.4, 160.3, 165.0; ESI-HRMS, *m*/*z*: calcd for C_8_H_12_BrO_3_S [M + H]^+^: 266.9685, found: 266.9683.

(4) 3-Bromo-5-isopropoxy-4-methylthiofuran-2(5*H*)-one (**3d**), yellowish oil, 94 mg, 88% (79% [32]); ^1^H NMR (600 MHz, CDCl_3_), *δ*, ppm: 1.31 (*d*, 3H, *J* = 6.0 Hz, CH_3_), 1.33 (*d*, 3H, *J* = 6.0 Hz, CH_3_), 2.60 (*s*, 3H, SCH_3_), 4.13–4.18 (*m*, 1H, OCH), 5.96 (*s*, 1H, CH); ^13^C NMR (150 MHz, CDCl_3_), *δ*, ppm: 13.2, 22.0, 23.2, 73.7, 100.5, 105.6, 160.3, 165.2.

(5) 3-Bromo-5-butoxy-4-methylthiofuran-2(5*H*)-one (**3e**), yellowish oil, 92 mg, 82%; ^1^H NMR (600 MHz, CDCl_3_), *δ*, ppm: 0.94 (*t*, *J* = 7.2 Hz, 3H, CH_3_), 1.38–1.44 (*m*, 2H, CH_2_), 1.62–1.67 (*m*, 2H, CH_2_), 2.60 (*s*, 3H, SCH_3_), 3.64–3.83 (*m*, 2H, OCH_2_), 5.93 (*s*, 1H, CH); ^13^C NMR (150 MHz, CDCl_3_), *δ*, ppm: 13.2, 13.7, 19.2, 31.4, 68.5, 101.4, 105.5, 160.2, 165.0; ESI-HRMS, *m*/*z*: calcd for C_9_H_14_BrO_3_S [M + H]^+^: 280.9842, found: 280.9837.

(6) 3-Bromo-4-methylthio-5-pentyloxyfuran-2(5*H*)-one (**3f**), yellowish oil, 93 mg, 79%; ^1^H NMR (600 MHz, CDCl_3_), *δ*, ppm: 0.92 (*t*, *J* = 6.0 Hz, 3H, CH_3_), 1.32–1.39 (*m*, 4H, 2CH_2_), 1.65–1.70 (*m*, 2H, CH_2_), 2.61 (*s*, 3H, SCH_3_), 3.64–3.84 (*m*, 2H, OCH_2_), 5.94 (*s*, 1H, CH); ^13^C NMR (150 MHz, CDCl_3_), *δ*, ppm: 13.2, 14.0, 22.3, 28.1, 29.0, 68.8, 101.4, 105.4, 160.3, 165.1; ESI-HRMS, *m*/*z*: calcd for C_10_H_16_BrO_3_S [M + H]^+^: 294.9998, found: 294.9998.

(7) 3-Bromo-5-heptyloxy-4-methylthiofuran-2(5*H*)-one (**3g**), yellowish oil, 84 mg, 76%; ^1^H NMR (600 MHz, CDCl_3_), *δ*, ppm: 0.89 (*t*, *J* = 7.2 Hz, 3H, CH_3_), 1.25–1.38 (*m*, 8H, 4CH_2_), 1.59–1.68 (*m*, 2H, CH_2_), 2.60 (*s*, 3H, SCH_3_), 3.63–3.82 (*m*, 2H, OCH_2_), 5.93 (*s*, 1H, CH); ^13^C NMR (150 MHz, CDCl_3_), *δ*, ppm: 13.2, 14.2, 22.6, 25.9, 28.9, 29.3, 31.7, 68.8, 101.4, 105.5, 160.3, 165.0; ESI-HRMS, *m*/*z*: calcd for C_12_H_20_BrO_3_S [M + H]^+^: 323.0311, found: 323.0305.

(8) 3-Bromo-5-cyclohexyloxy-4-methylthiofuran-2(5*H*)-one (**3h**), yellowish oil, 95 mg, 78% (78% [32]); ^1^H NMR (600 MHz, CDCl_3_), *δ*, ppm: 1.19–1.59 (*m*, 6H, 3CH_2_), 1.74–2.01 (*m*, 4H, 2CH_2_), 2.60 (*s*, 3H, SCH_3_), 3.81–3.86 (*m*, 1H, OCH), 6.00 (*s*, H, CH); ^13^C NMR (150 MHz, CDCl_3_), *δ*, ppm: 13.3, 23.9, 24.0, 25.3, 32.1, 33.2, 79.3, 100.4, 105.5, 160.5, 165.3.

(9) 5-Benzyloxy-3-bromo-4-methylthiofuran-2(5*H*)-one (**3i**), yellowish oil, 93 mg, 74% (75% [32]); ^1^H NMR (600 MHz, CDCl_3_), *δ*, ppm: 2.49 (*s*, 3H, SCH_3_), 4.70–4.87 (*m*, 2H, OCH_2_), 5.97 (*s*, 1H, CH), 7.36–7.41 (*m*, 5H, ArH); ^13^C NMR (150 MHz, CDCl_3_), *δ*, ppm: 13.2, 70.5, 100.0, 105.5, 128.8, 128.9, 134.9, 160.5, 165.0.

(10) 3-Bromo-5-menthoxy-4-methylthiofuran-2(5*H*)-one (**3j**), yellowish oil, 107 mg, 74% (71% [32]); ^1^H NMR (600 MHz, CDCl_3_), *δ*, ppm: 0.85–0.91 (*m*, 10H, CH, 3CH_3_), 1.24–1.34 (*m*, 3H, CH, CH_2_), 1.57–1.73 (*m*, 3H, CH, CH_2_), 1.89–2.31 (*m*, 2H, CH_2_), 2.63 (*s*, 3H, SCH_3_), 3.99–4.03 (*m*, 1H, OCH), 5.88 (*s*, 1H, CH); ^13^C NMR (150 MHz, CDCl_3_), *δ*, ppm: 13.6, 14.1, 18.8, 19.6, 26.6, 28.0, 37.1, 44.9, 47.7, 49.5, 88.0, 102.9, 106.0, 160.5, 165.2. 

(11) 5-([1,1′-Biphenyl]-4-yloxy)-3-bromo-4-methylthiofuran-2(5*H*)-one (**3k**), yellowish oil, 69 mg, 46% (39% [32]); ^1^H NMR (600 MHz, CDCl_3_), *δ*, ppm: 2.66 (*s*, 3H, SCH_3_), 6.41 (*s*, 1H, CH), 7.26 (*d*, *J* = 9.0 Hz, 2H, ArH), 7.38 (*t*, *J* = 7.2 Hz, 1H, ArH), 7.42–7.52 (*m*, 2H, ArH), 7.58 (*d*, *J* = 8.4 Hz, 2H, ArH), 7.61 (*d*, *J* = 8.4 Hz, 2H, ArH); ^13^C NMR (150 MHz, CDCl_3_), *δ*, ppm: 13.6, 99.5, 106, 117.2, 127.0, 127.4, 128.7, 128.9, 137.6, 140.1, 154.9, 159.8, 164.6.

(12) 3-Chloro-5-methoxy-4-methylthiofuran-2(5*H*)-one (**3l**), yellowish oil, 56 mg, 72% (60% [32]); ^1^H NMR (600 MHz, CDCl_3_), *δ*, ppm: 2.61 (*s*, 3H, SCH_3_), 3.54 (*s*, 3H, OCH_3_), 5.89 (*s*, 1H, CH); ^13^C NMR (150 MHz, CDCl_3_), *δ*, ppm: 13.1, 55.0, 100.9, 117.1, 155.4, 164.4.

(13) 3-Chloro-5-ethoxy-4-methylthiofuran-2(5*H*)-one (**3m**), yellowish oil, 52 mg, 63% (58% [32]); ^1^H NMR (600 MHz, CDCl_3_), *δ*, ppm: 1.31 (*t*, *J* = 7.2 Hz, 3H, CH_3_), 2.60 (*s*, 3H, SCH_3_), 3.73–3.92 (*m*, 2H, OCH_2_), 5.93 (*s*, 1H, CH); ^13^C NMR (150 MHz, CDCl_3_), *δ*, ppm: 13.2, 15.0, 64.6, 101.3, 105.5, 160.2, 165.0.

(14) 3-Chloro-4-methylthio-5-propoxyfuran-2(5*H*)-one (**3n**), yellowish oil, 55 mg, 62%; ^1^H NMR (600 MHz, CDCl_3_), *δ*, ppm: 0.97 (*t*, *J* = 6.0 Hz, 3H, CH_3_), 1.66–1.73 (*m*, 2H, CH_2_), 2.61 (*s*, 3H, SCH_3_), 3.60–3.81 (*m*, 2H, OCH_2_), 5.92 (*s*, 1H, CH); ^13^C NMR (150 MHz, CDCl_3_), *δ*, ppm: 10.5, 13.1, 22.7, 70.5, 100.4, 117.0, 155.7, 164.6; ESI-HRMS, *m*/*z*: calcd for C_8_H_12_ClO_3_S [M + H]^+^: 223.0190, found: 223.0187.

(15) 3-Chloro-5-isopropoxy-4-methylthiofuran-2(5*H*)-one (**3o**), yellowish oil, 50 mg, 56% (55% [32]); ^1^H NMR (600 MHz, CDCl_3_), *δ*, ppm: 1.31 (*d*, 3H, *J* = 6.0 Hz, CH_3_), 1.33 (*d*, 3H, *J* = 6.0 Hz, CH_3_), 2.61 (*s*, 3H, SCH_3_), 4.12–4.17 (*m*, 1H, OCH), 5.94 (*s*, 1H, CH); ^13^C NMR (150 MHz, CDCl_3_), *δ*, ppm: 13.1, 22.0, 23.2, 73.8, 99.5, 117.1, 155.8, 164.8.

(16) 5-Butoxy-3-chloro-4-methylthiofuran-2(5*H*)-one (**3p**), yellowish oil, 67 mg, 71%; ^1^H NMR (600 MHz, CDCl_3_), *δ*, ppm: 0.94 (*t*, *J* = 7.2 Hz, 3H, CH_3_), 1.37–1.45 (*m*, 2H, CH_2_), 1.62–1.68 (*m*, 2H, CH_2_), 2.61 (*s*, 3H, SCH_3_), 3.64–3.85 (*m*, 2H, OCH_2_), 5.91 (*s*, 1H, CH); ^13^C NMR (150 MHz, CDCl_3_), *δ*, ppm: 13.1, 13.7, 19.1, 31.4, 68.7, 100.4, 117.0, 155.6, 164.6; ESI-HRMS, *m*/*z*: calcd for C_9_H_14_ClO_3_S [M + H]^+^: 237.0347, found: 237.0346.

(17) 3-Chloro-4-methylthio-5-pentyloxyfuran-2(5*H*)-one (**3q**), yellowish oil, 69 mg, 69%; ^1^H NMR (600 MHz, CDCl_3_), *δ*, ppm: 0.91 (*t*, *J* = 6.0 Hz, 3H, CH_3_), 1.33–1.38 (*m*, 4H, 2CH_2_), 1.64–1.69 (*m*, 2H, CH_2_), 2.61 (*s*, 3H, SCH_3_), 3.63–3.84 (*m*, 2H, OCH_2_), 5.91 (*s*, 1H, CH); ^13^C NMR (150 MHz, CDCl_3_), *δ*, ppm: 13.1, 13.9, 22.3, 28.1, 29.0, 69.0, 100.4, 117.0, 155.6, 164.6; ESI-HRMS, *m*/*z*: calcd for C_10_H_16_ClO_3_S [M + H]^+^: 251.0503, found: 251.0502.

(18) 3-Chloro-5-cyclohexyloxy-4-methylthiofuran-2(5*H*)-one (**3r**), yellowish oil, 54 mg, 52% (51% [32]); ^1^H NMR (600 MHz, CDCl_3_), *δ*, ppm: 1.22–1.50 (*m*, 6H, 3CH_2_), 1.75–2.01 (*m*, 4H, 2CH_2_), 2.61 (*s*, 3H, SCH_3_), 3.80–3.85 (*m*, 1H, OCH), 5.98 (*s*, 1H, CH); ^13^C NMR (150 MHz, CDCl_3_), *δ*, ppm: 13.2, 23.9, 24.0, 25.3, 32.1, 33.2, 79.3, 99.4, 117.1, 155.9, 164.8.

(19) 5-Benzyloxy-3-chloro-4-methylthiofuran-2(5*H*)-one (**3s**), yellowish oil, 54 mg, 50% (52% [32]); ^1^H NMR (600 MHz, CDCl_3_), *δ*, ppm: 2.52 (*s*, 3H, SCH_3_), 4.71–4.88 (*m*, 2H, OCH_2_), 5.96 (*s*, 1H, CH), 7.37–7.41 (*m*, 5H, ArH); ^13^C NMR (150 MHz, CDCl_3_), *δ*, ppm: 13.2, 70.6, 98.9, 117.1, 128.8, 128.9, 135.0, 155.8, 164.5.

(20) 3-Chloro-4-methylthio-5-phenoxyfuran-2(5*H*)-one (**3t**), yellowish oil, 50 mg, 49% (46% [32]); ^1^H NMR (600 MHz, CDCl_3_), *δ*, ppm: 2.63 (*s*, 3H, SCH_3_), 6.35 (*s*, 1H, CH), 7.15–7.18 (*m*, 3H, ArH), 7.35–7.39 (*m*, 2H, ArH); ^13^C NMR (150 MHz, CDCl_3_), *δ*, ppm: 13.4, 98.5, 116.9, 124.4, 130.0, 155.3, 155.6, 164.1.

The detailed ^1^H, ^13^C NMR and spectra for all compounds **3a**–**3t** are provided in the Supplementary Materials.

## 4. Conclusions

In conclusion, we have disclosed a methylthiolating reaction of 5-alkoxy (or 5-aryoxy)-substituted 3,4-dihalo-2(5*H*)-furanone with DMSO. DMSO cannot only be used as a reaction raw material, but also as a solvent for the reaction. In addition, this transformation without any transition metal catalysts only requires a lower temperature and a shorter time. In particular, the simple reaction system is easy to operate, giving a better yield, even for the gram-scale reaction. This successful investigation provides a valuable reference for the introduction of the methyl sulfur group in organic synthesis.

Notably, due to the marked acetal and lactone ring structure of the reaction substrate with different functional groups such as C-X (X = Cl or Br), C-O, C=O and C=C bonds, the simple and green method will be attractive for synthesizing potentially bioactive methyl sulfur compounds with multifunctional groups.

## Figures and Tables

**Table 1:** Optimization of reaction conditions ^[a]^.

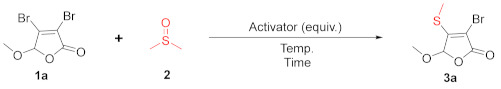				
Entry	Activator (Equiv.)	Temp. (°C)	Time (h)	Yield of 3a (%) ^[b]^
1	NBS (1.0)	80	5	65
2	NCS (1.0)	80	5	43
3	DBDMH (1.0)	80	5	87
4	DBDMH (0.5)	80	5	78
**5**	**DBDMH (1.5)**	**80**	**5**	**93**
6	DBDMH (2.0)	80	5	90
7	DBDMH (1.5)	60	5	85
8	DBDMH (1.5)	100	5	87
9	DBDMH (1.5)	80	8	89
10	DBDMH (1.5)	80	3	57

^[a]^ Reaction conditions: **1a** (0.4 mmol) and **2** (2 mL) were added and stirred for 5 h. ^[b]^ Isolated yield.

**Table 2:** Substrate scope of various 5-substituted-3,4-dihalo-2(5*H*)-furanone intermediate **1** ^[a,b]^ [32].

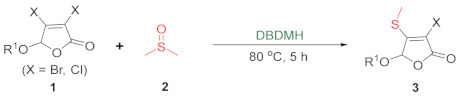
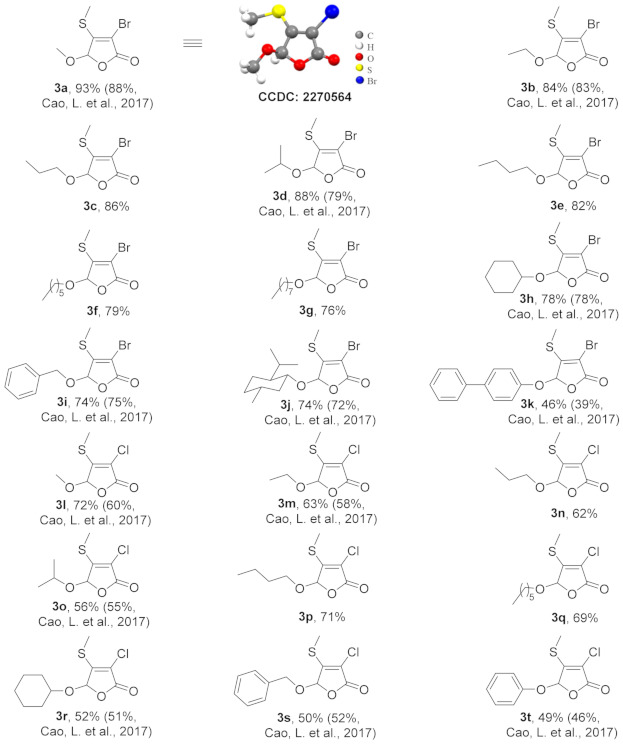

^[a]^ Reaction conditions: **1** (0.4 mmol), DBDMH (0.6 mmol), **2** (2 mL) were added and stirred at 80 °C for 5 h. ^[b]^ Isolated yield.

**Table 3:** Crystal data and structure refinement for **3a**.

Compound	3a
Empirical formula	C_6_H_7_BrO_3_S
Formula weight	239.09
Temperature (K)	297
Wavelength (Å)	0.71073
Crystal system	Monoclinic
Space group	P2_1_
Unit cell dimensions (Å, °)	*a* = 4.1083 (8), *b* = 7.6804 (19), *c* = 13.739 (3) *α* = 90, *β* = 90.079 (17), *γ* = 90
Volume (Å^3^)	433.22 (16)
Z	2
Density (calculated) (g/cm^3^)	1.833
Absorption coefficient (mm^−1^)	4.941
F(000)	236.0
Theta range for data collection	3.981 to 22.995
Index ranges	−4 ≤ h ≤ 4, −9 ≤ k ≤ 9, 0 ≤ l ≤ 16
Reflections collected	1464
Independent reflections	1464 [R_sigma_ = 0.1075]
Completeness to theta = 1.78°	99.6%
Absorption correction	Multiscan
Max. and min. transmission	1.000 and 0.101
Refinement method	Least squares minimization
Data/restraints/parameters	1464/1/103
Goodness-of-fit on F^2^	1.040
Final R indices [I > 2 sigma (I)]	R_1_ = 0.0545, wR_2_ = 0.0955
R indices (all data)	R_1_ = 0.0707, wR_2_ = 0.1048
Largest diff. peak and hole	0.58 and −0.84 e.Å^−3^

## Data Availability

All data supporting the findings of this study are available within the paper and within its Supplementary Materials published online.

## Supplementary Materials

The following supporting information can be downloaded at https://www.mdpi.com/article/10.3390/molecules28155635/s1, which contains the ^1^H and ^13^C NMR spectra for all compounds **3a**–**3t**.
