# Supplementary material for: DBDMH-Promoted Methylthiolation in DMSO: A Metal-Free Protocol to Methyl Sulfur Compounds with Multifunctional Groups
Source: Molecules. 2023 Jul 25;28(15):5635. doi: 10.3390/molecules28155635 (PMC10419854; doi:10.3390/molecules28155635)
Supplement: Supplementary file 1 [file molecules-28-05635-s001.zip › molecules-2497203-supplementary.pdf]

Supplementary Materials

DBDMH-promoted Methylthiolation in DMSO: A Metal-free Protocol to Methyl Sulfur Compounds with Multifunctional Groups

Yong-Jun Zhou [1], Yong-Gan Fang [1], Kai Yang [1,2,*], Jian-Yun Lin [1,3,], Huan-Qing Li [1], Zu-Jia Chen [1] and Zhao-Yang Wang [1,*]

1 School of Chemistry, South China Normal University; GDMPA Key Laboratory for Process Control and Quality Evaluation of Chiral Pharmaceuticals; Guangzhou Key Laboratory of Analytical Chemistry for Biomedicine; Key Laboratory of Theoretical Chemistry of Environment, Ministry of Education, Guang-zhou, Guangdong 510006, P. R. China; 2020022483@m.scnu.edu.cn (Y.-J.Z.); 2020022476@m.scnu.edu.cn (Y.-G.F.); jianyunlin@outlook.com (J.-Y.L.); 2022022611@m.scnu.edu.cn (H.-Q.L.); chenzujia2022@163.com (Z.-J.C.)

2 College of Pharmacy, Gannan Medical University, Ganzhou 341000, P. R. China

3 School of Mechanical and Automotive Engineering, South China University of Technology, Guangzhou 510640, China

* Correspondence: wangzy@scnu.edu.cn (Z.-Y.W.); kai_yangyang@126.com (K.Y.); Fax: (+86)-020-3931-0187; Tel: (+86)-020-3931-0258.

Table of Contents

^{1}H and ^{13}C NMR Spectra for All Compounds **3a-3t**..[2-21]

NMR Spectra for All Compounds 3a-3t

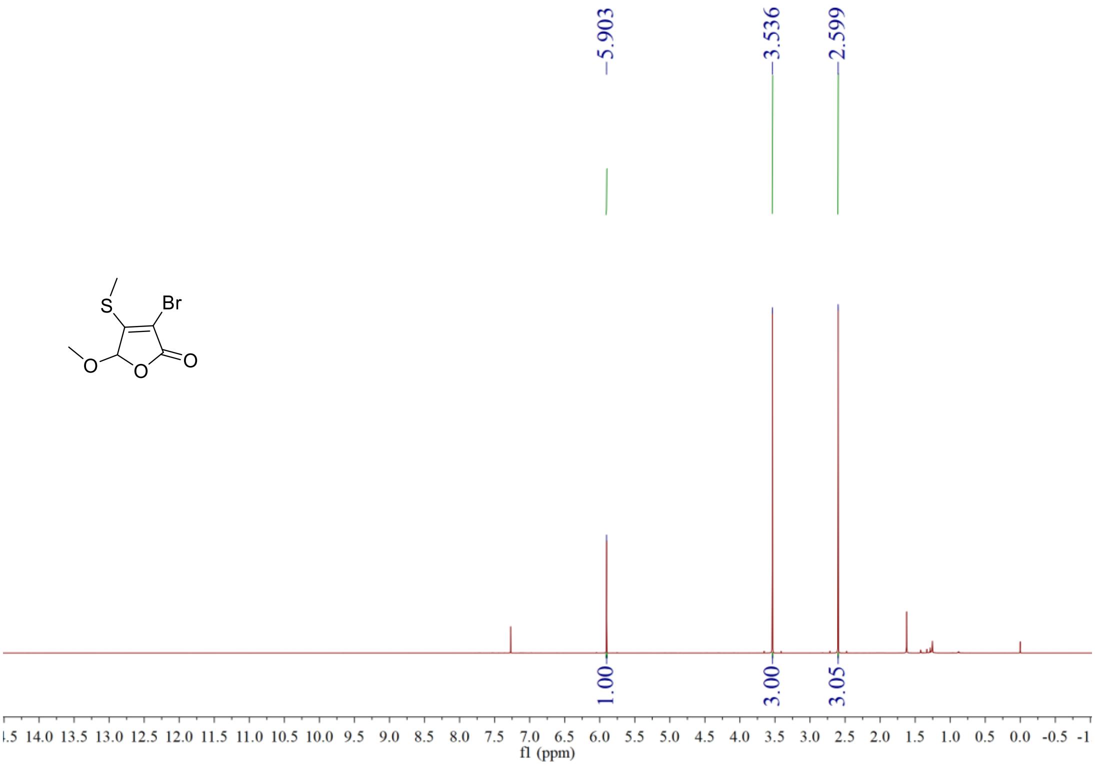

^{1}H NMR spectrum of compound **3a**

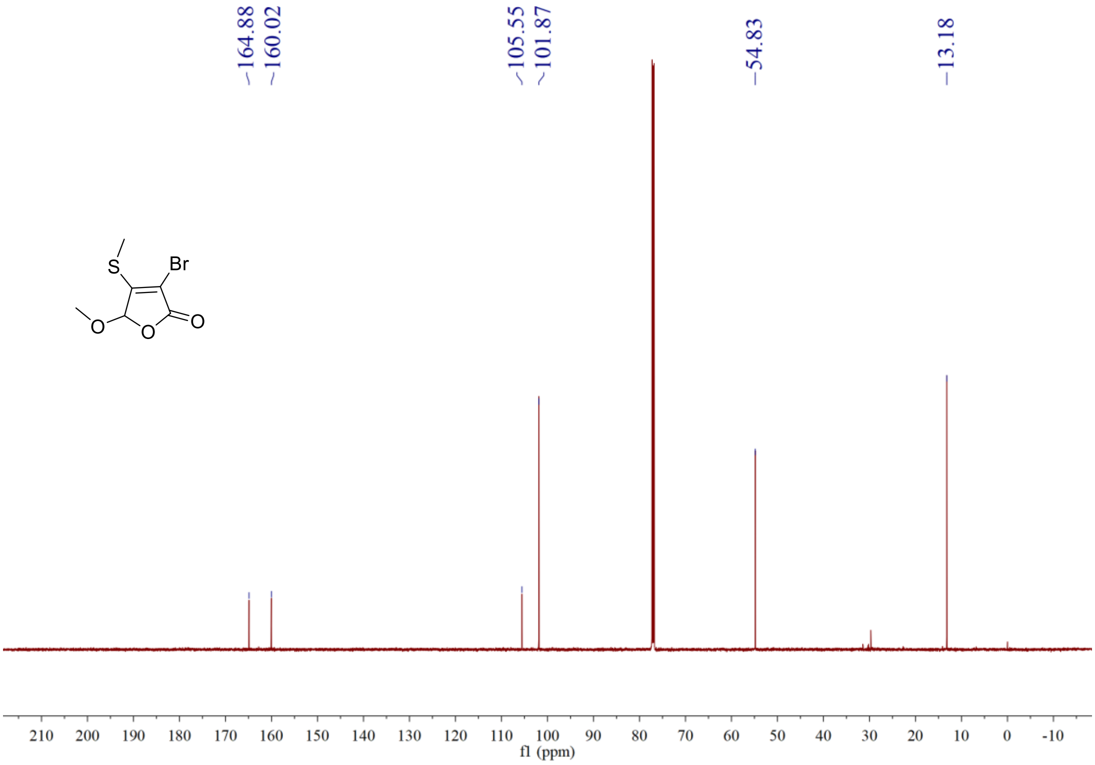

^{13}C NMR spectrum of compound **3a**

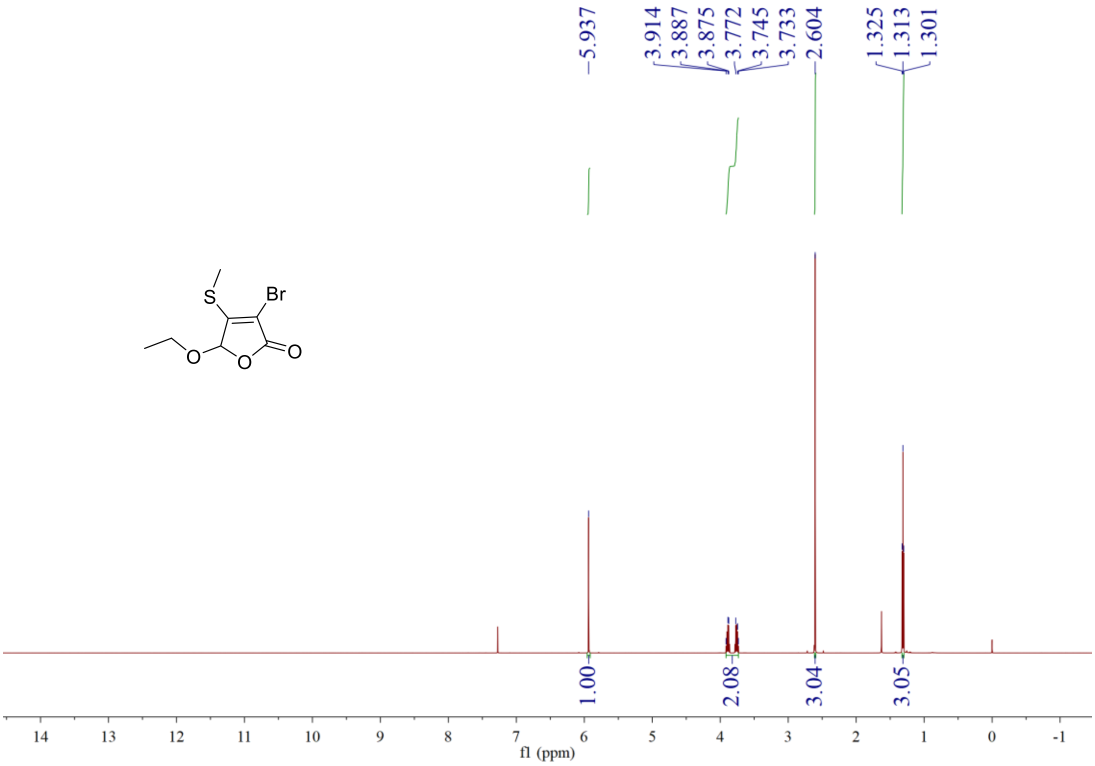

^{1}H NMR spectrum of compound **3b**

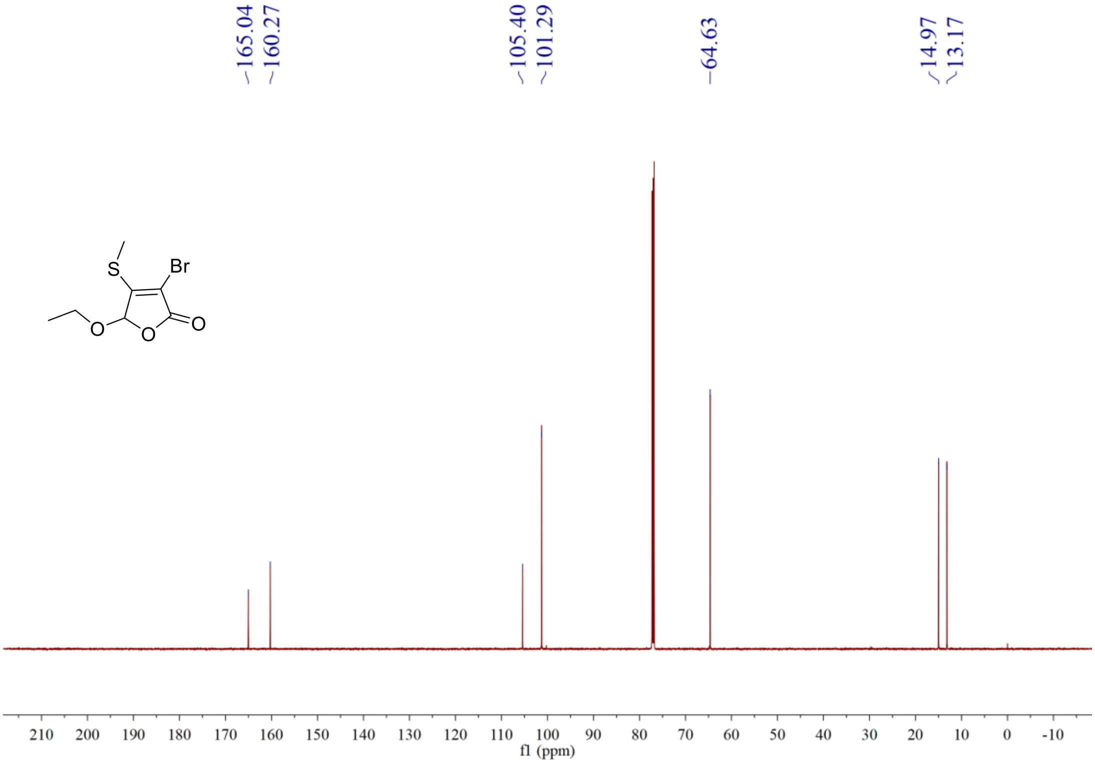

^{13}C NMR spectrum of compound **3b**

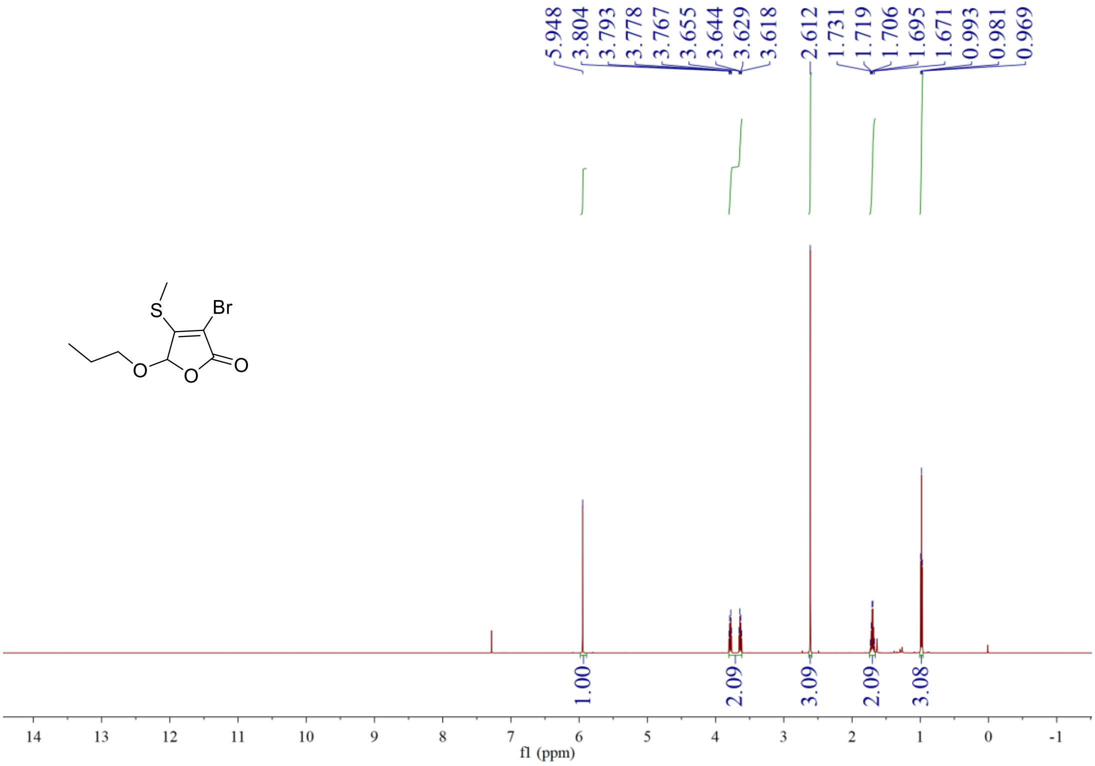

^{1}H NMR spectrum of compound **3c**

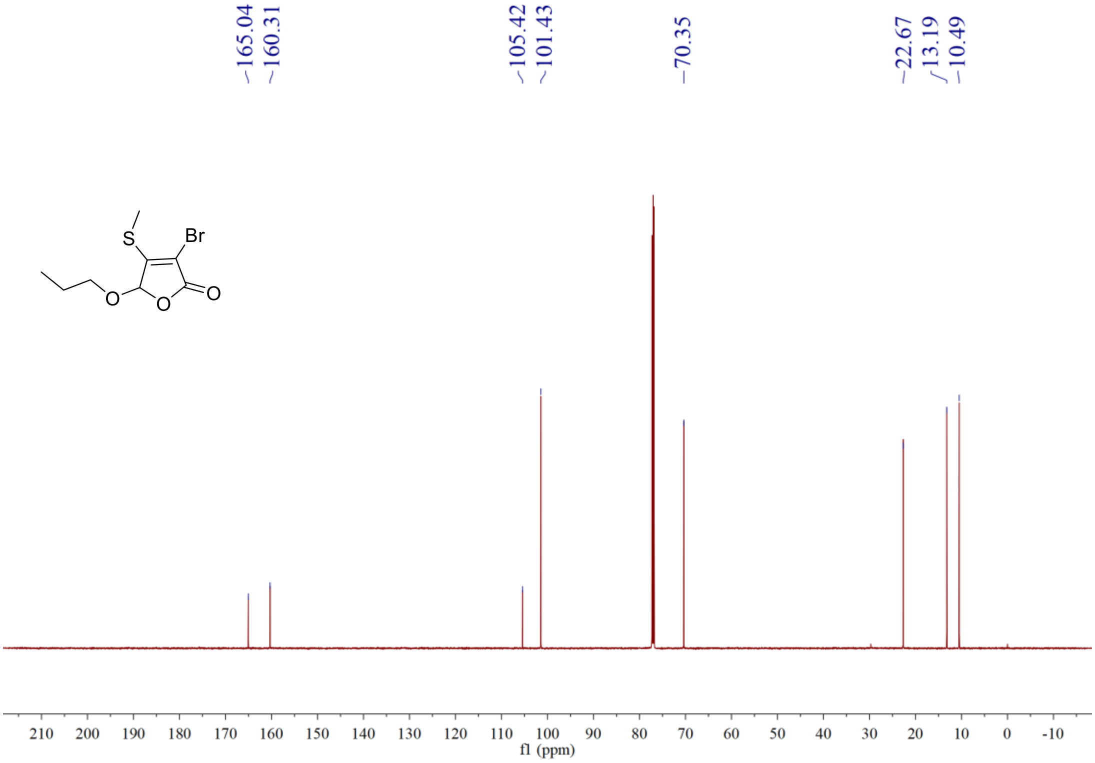

^{13}C NMR spectrum of compound **3c**

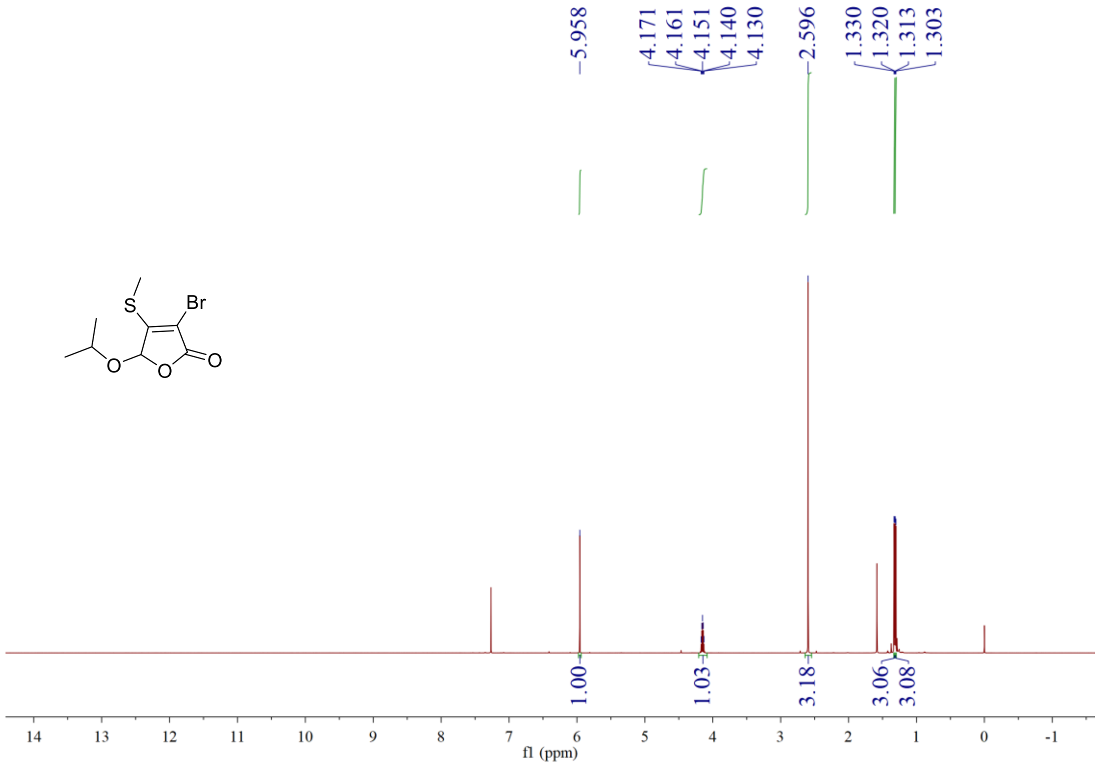

^{1}H NMR spectrum of compound **3d**

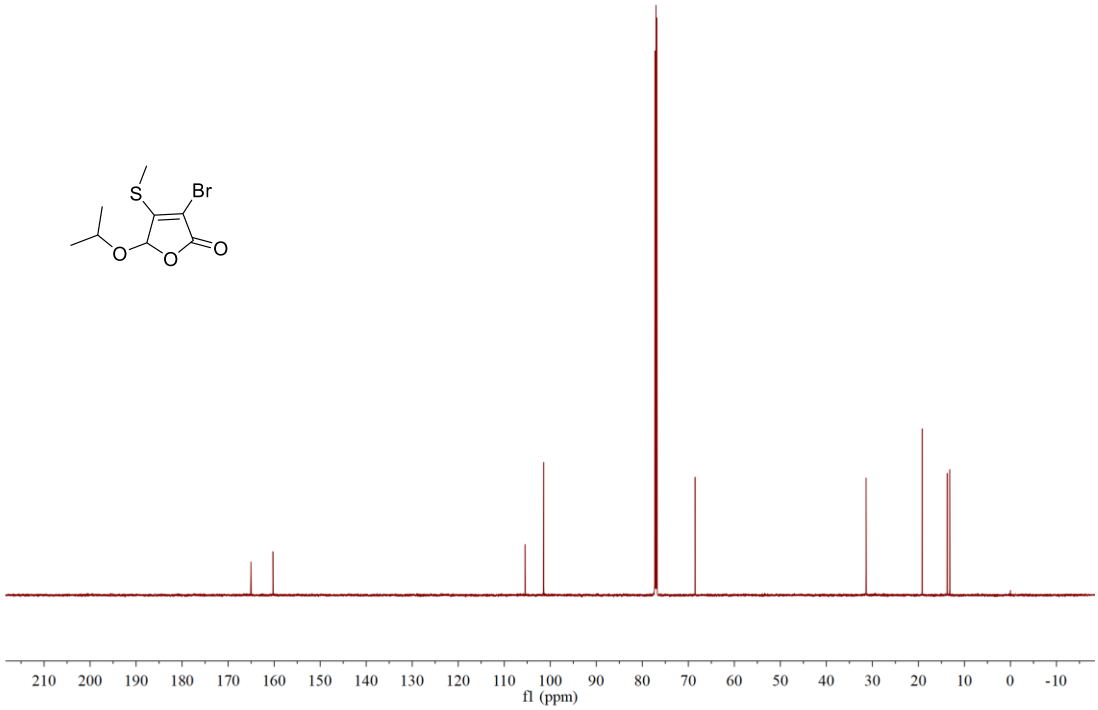

^{13}C NMR spectrum of compound **3d**

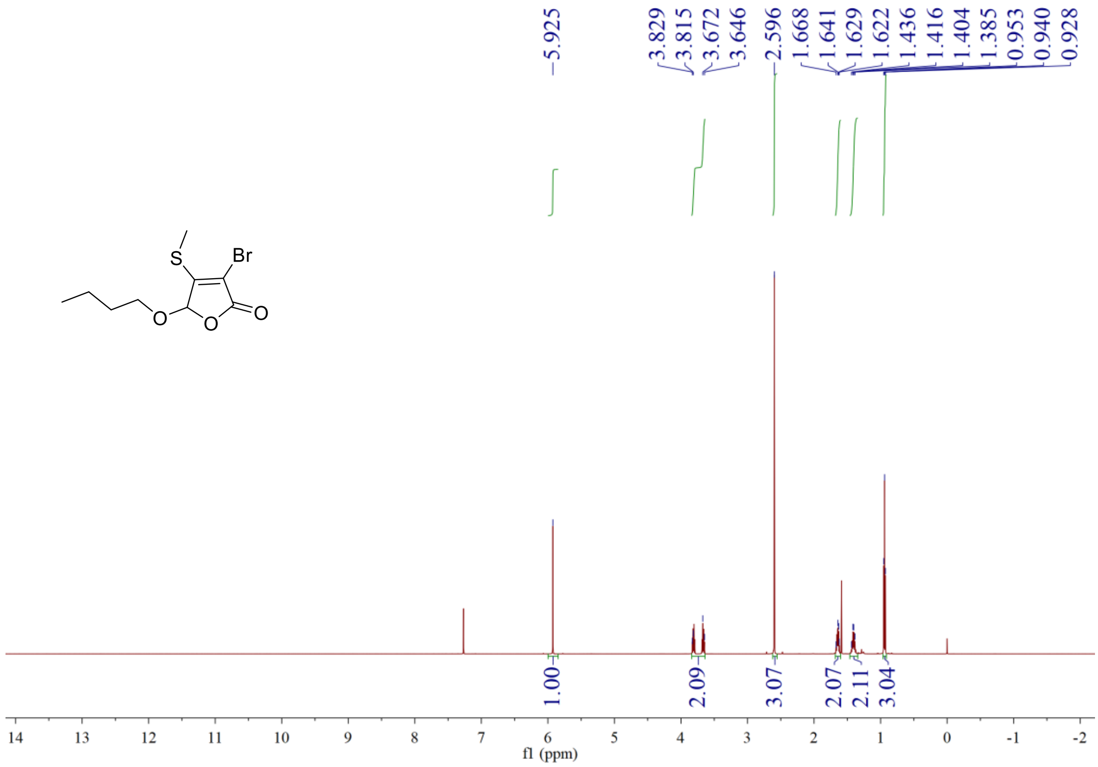

^{1}H NMR spectrum of compound **3e**

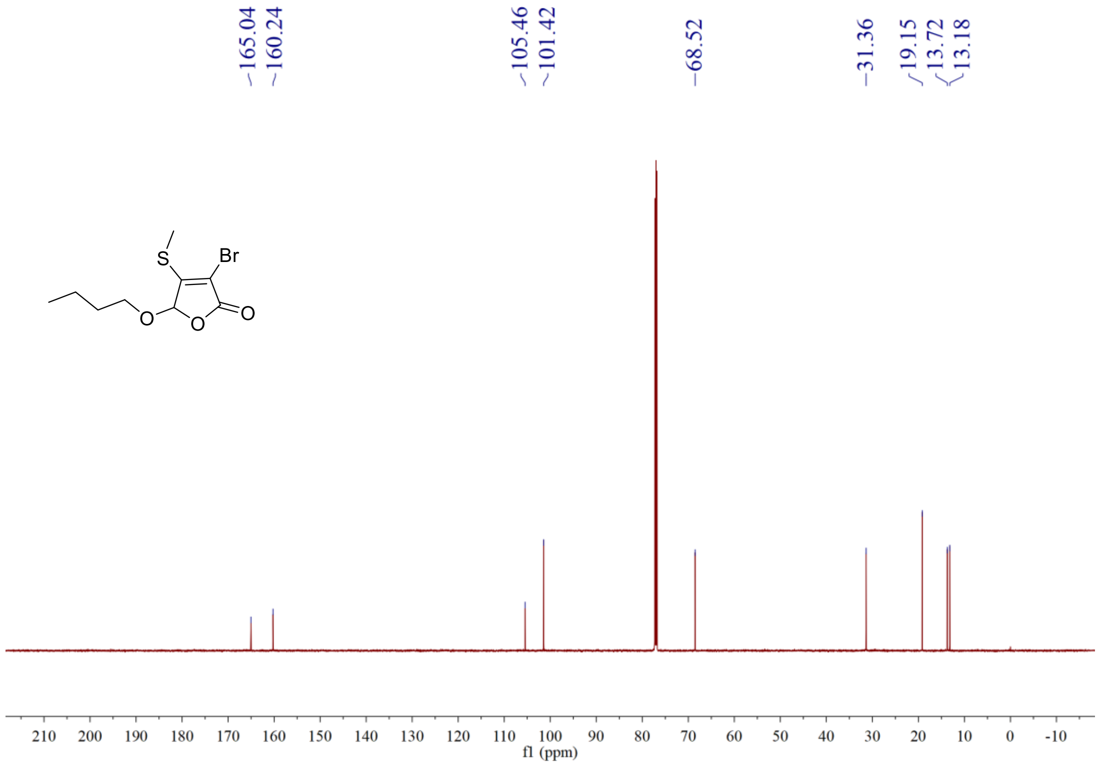

^{13}C NMR spectrum of compound **3e**

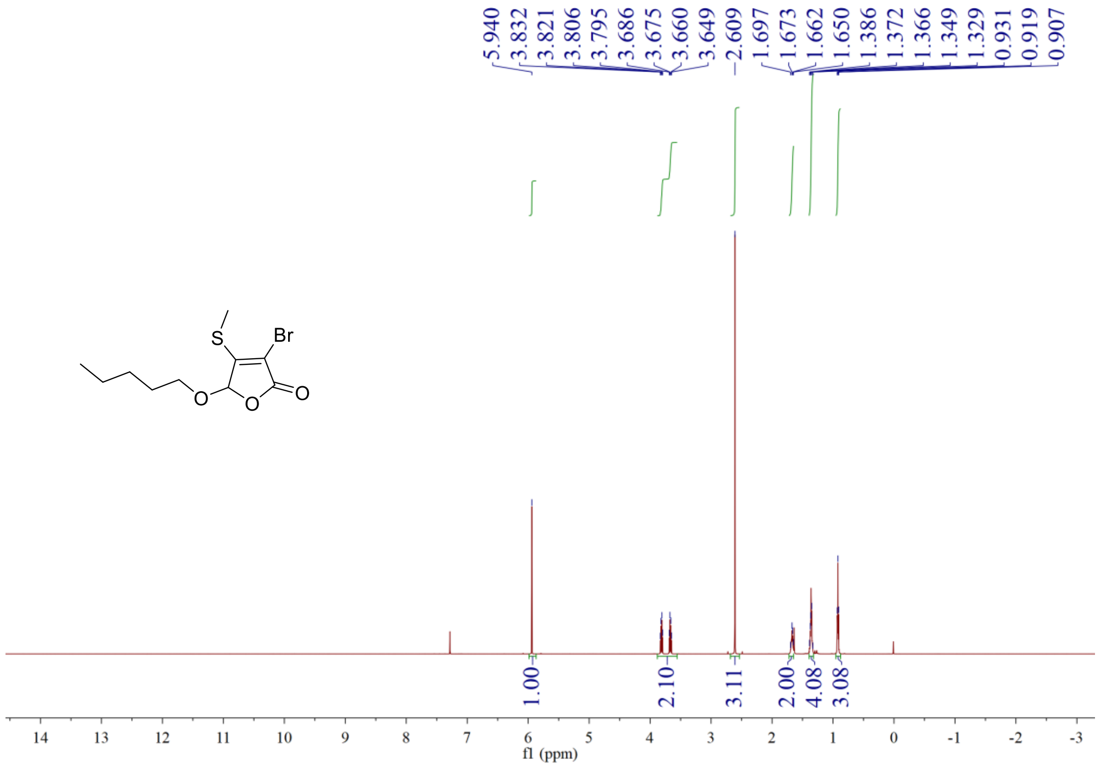

^{1}H NMR spectrum of compound **3f**

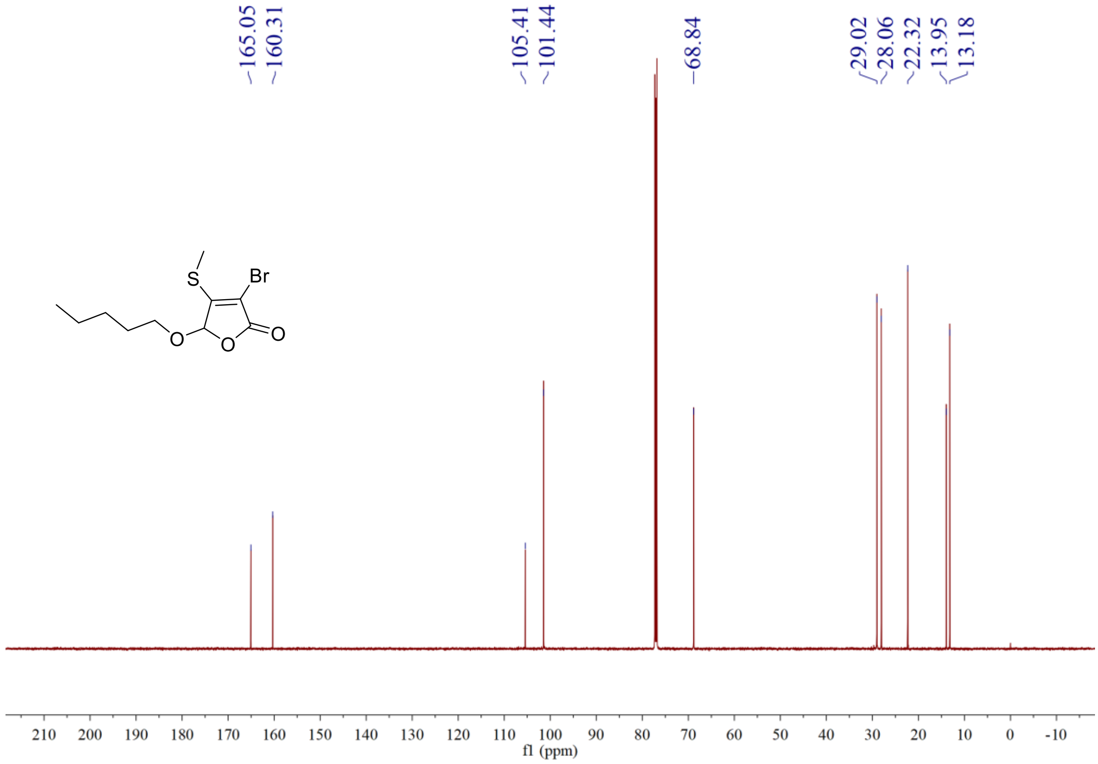

^{13}C NMR spectrum of compound **3f**

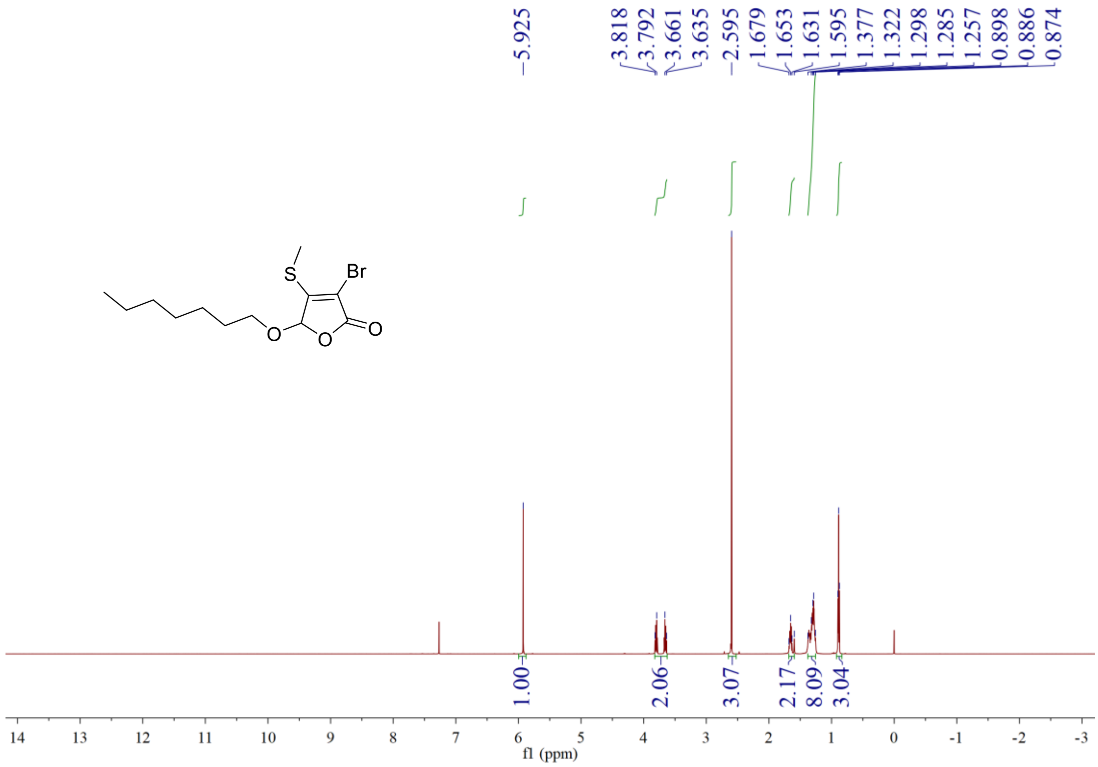

^{1}H NMR spectrum of compound **3g**

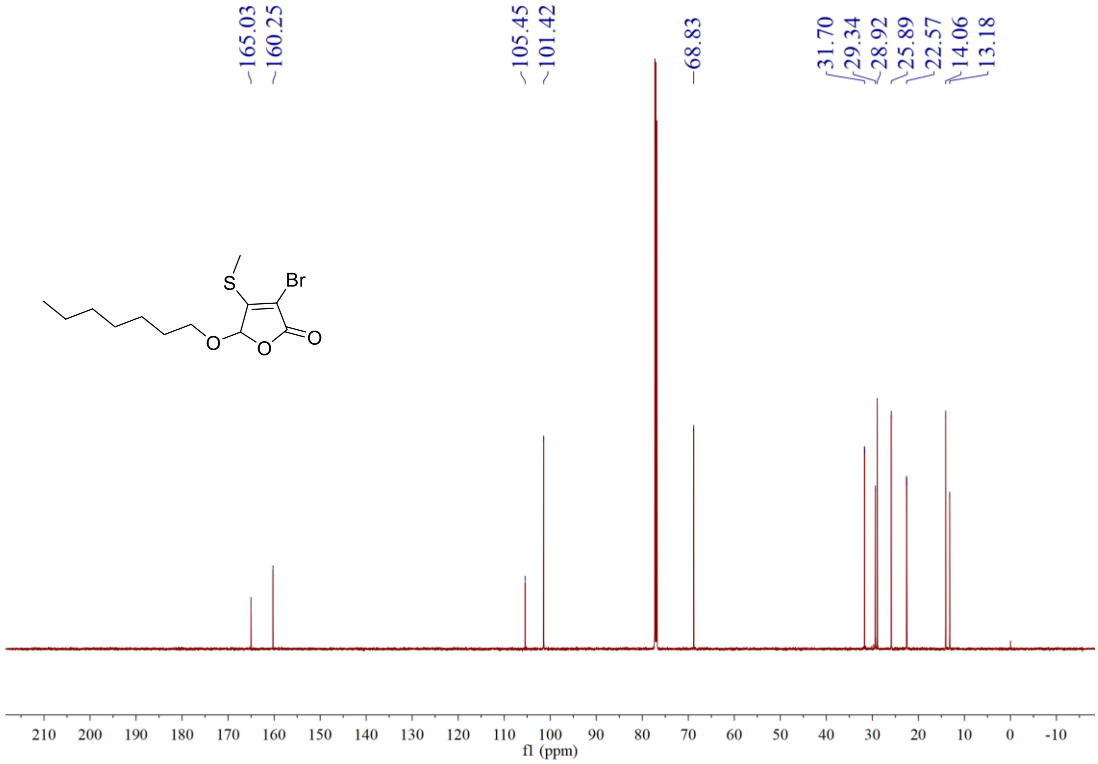

^{13}C NMR spectrum of compound **3g**

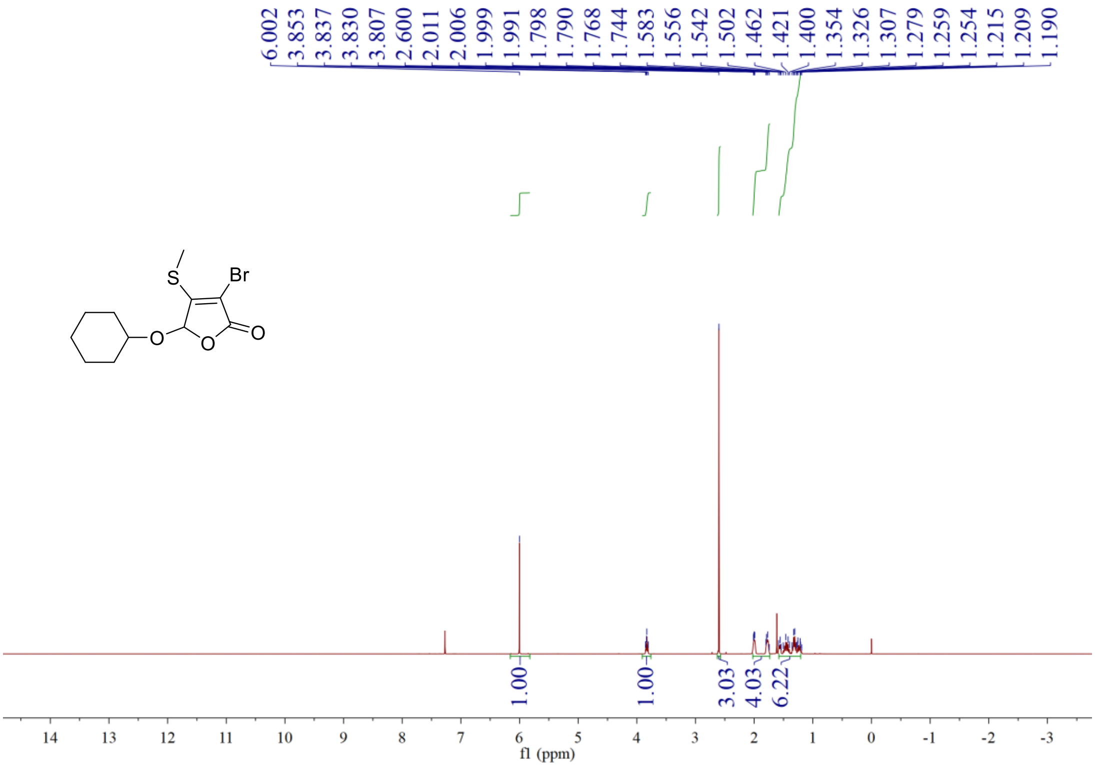

^{1}H NMR spectrum of compound **3h**

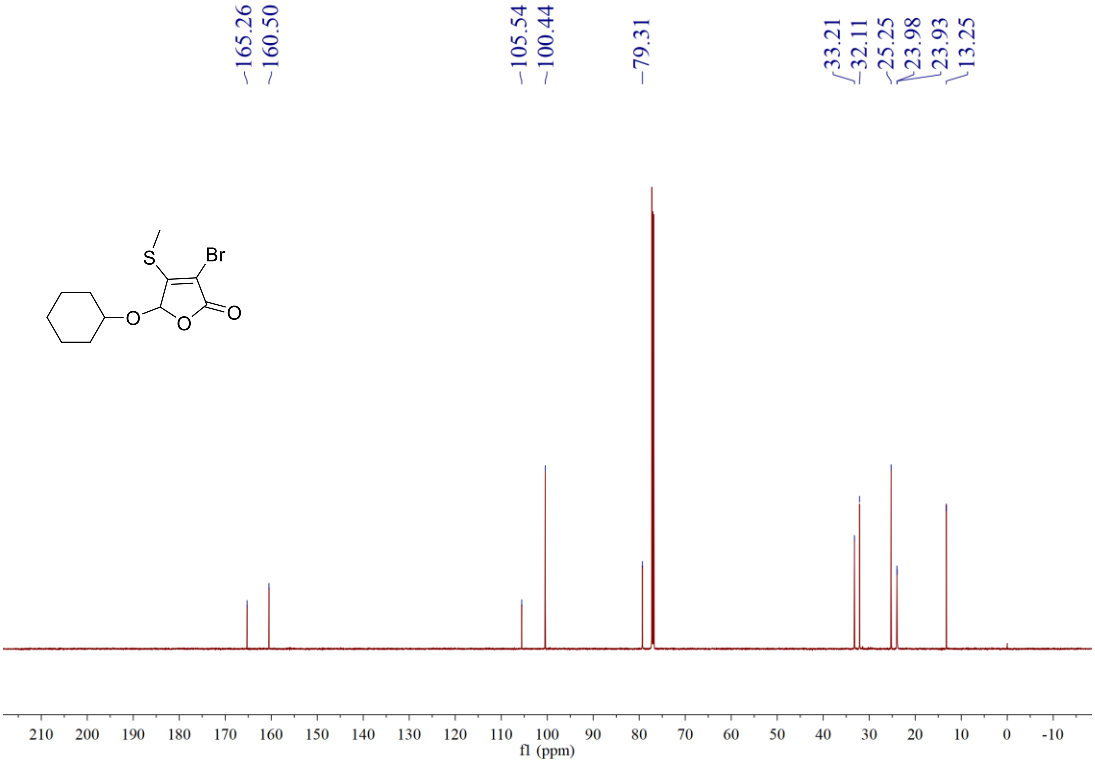

^{13}C NMR spectrum of compound **3h**

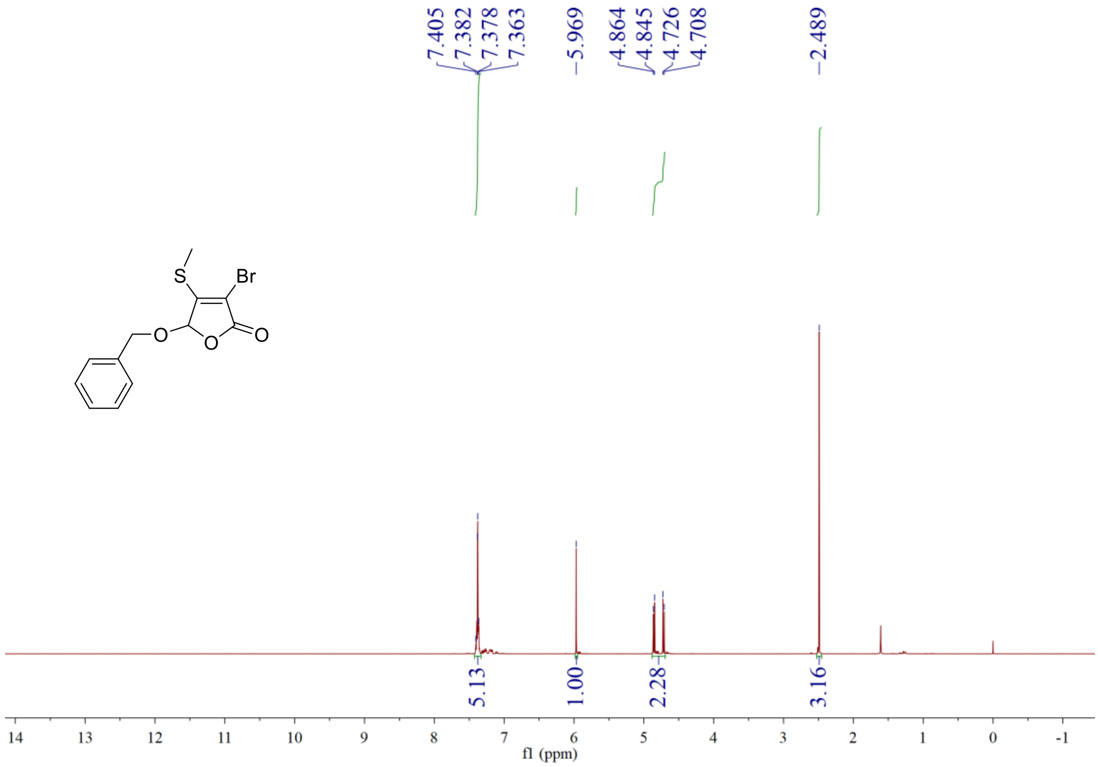

^{1}H NMR spectrum of compound **3i**

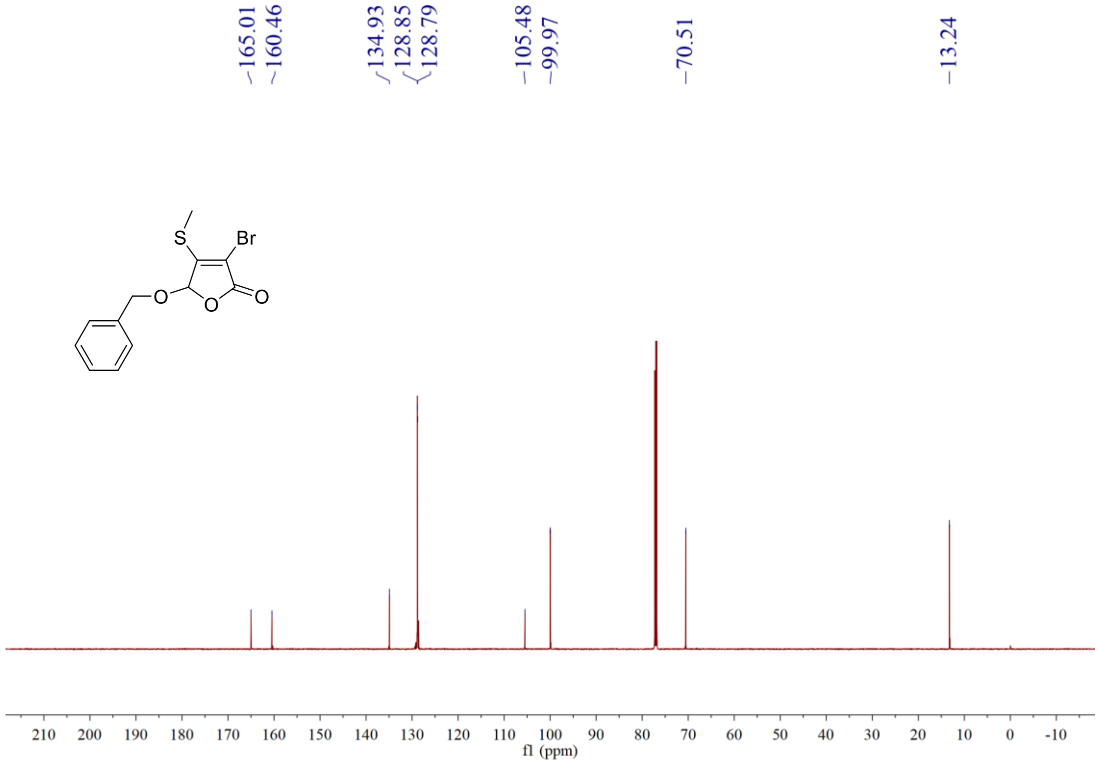

^{13}C NMR spectrum of compound **3i**

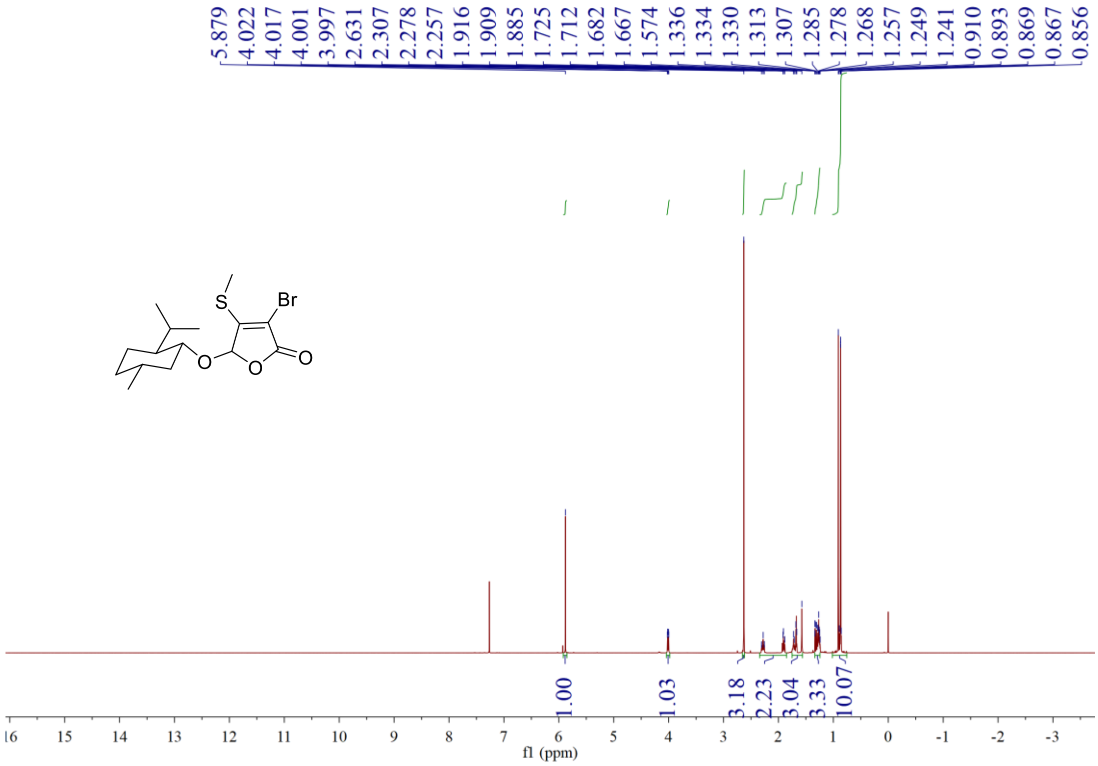

^{1}H NMR spectrum of compound **3j**

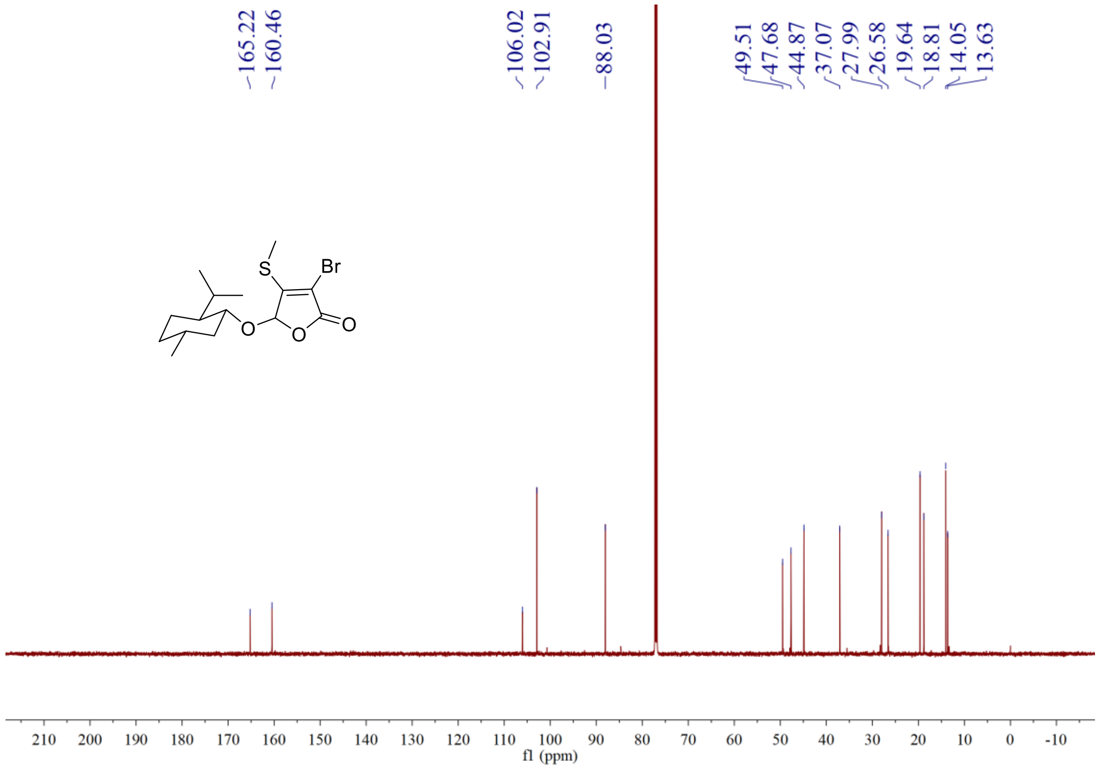

^{13}C NMR spectrum of compound **3j**

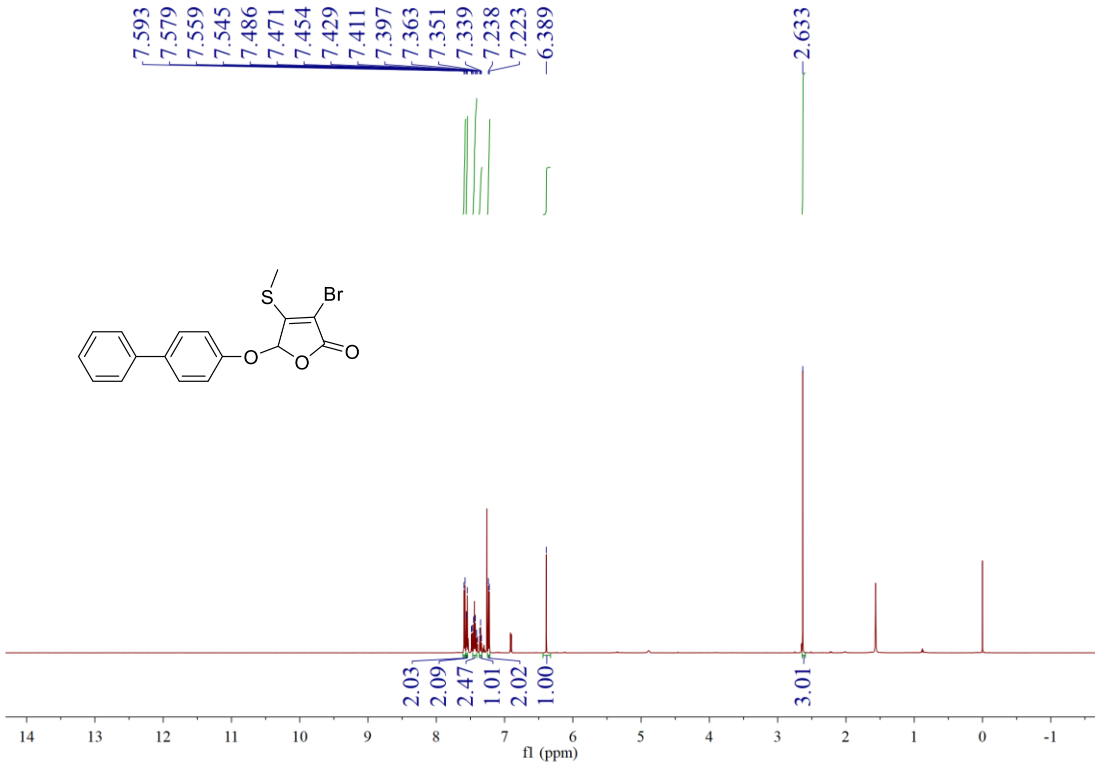

^{1}H NMR spectrum of compound **3k**

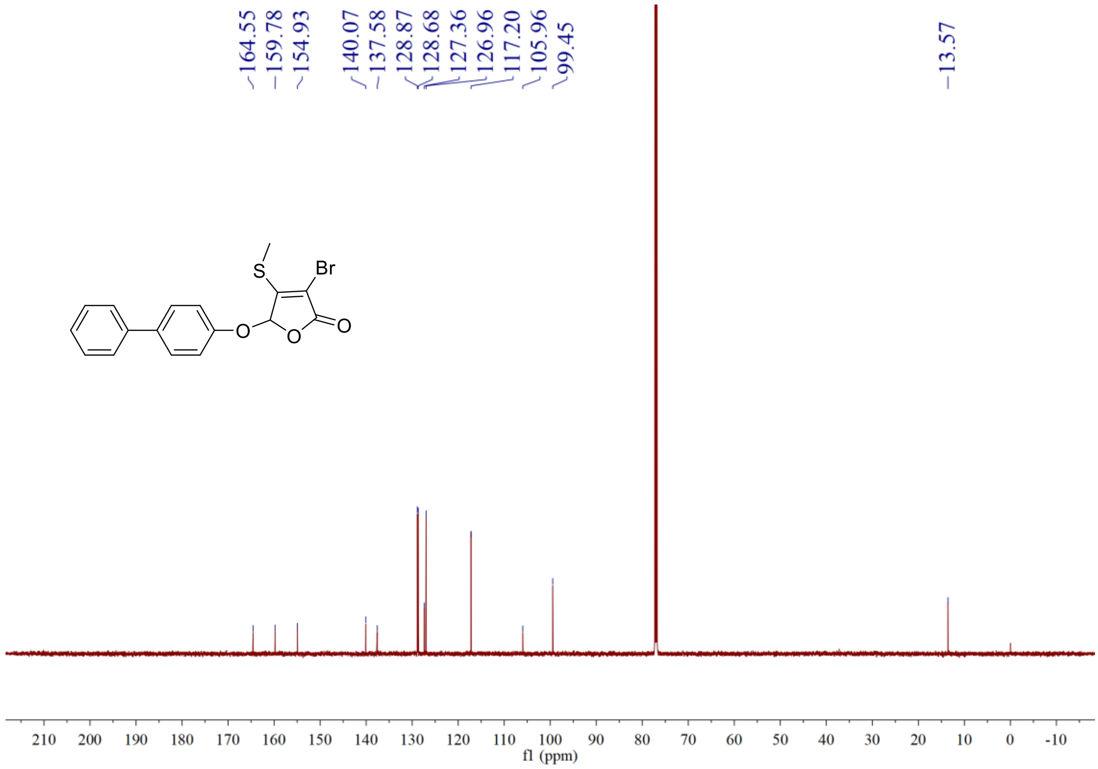

^{13}C NMR spectrum of compound **3k**

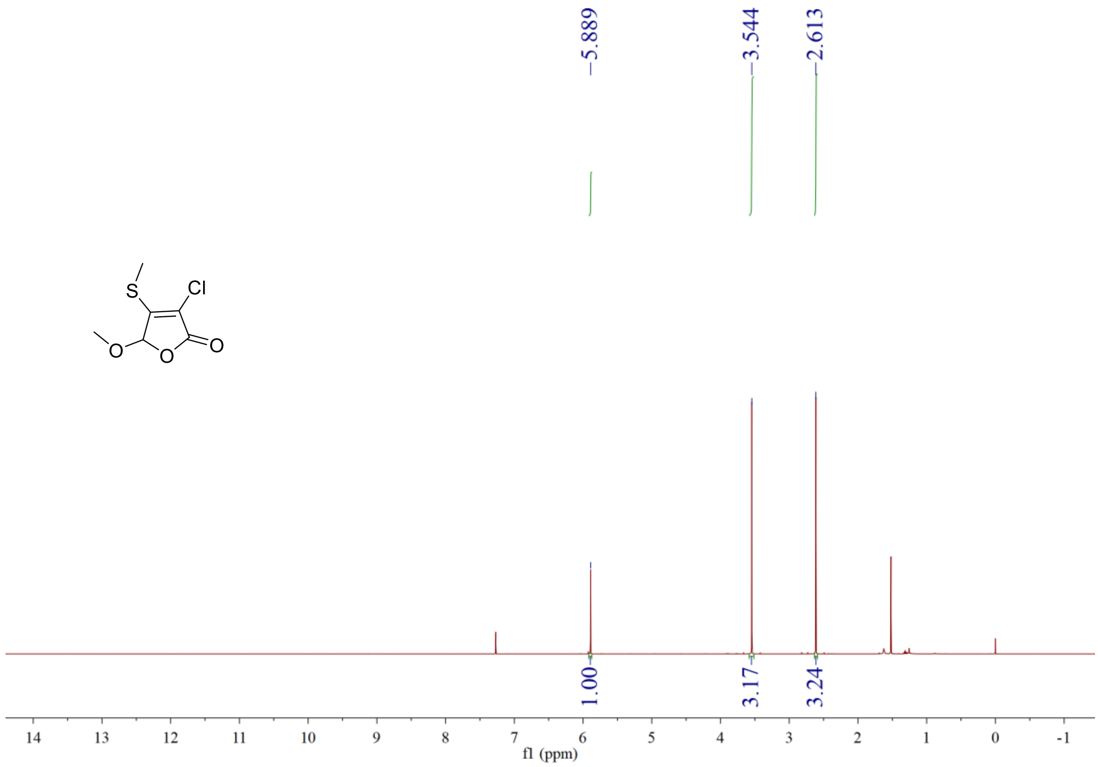

^{1}H NMR spectrum of compound **3l**

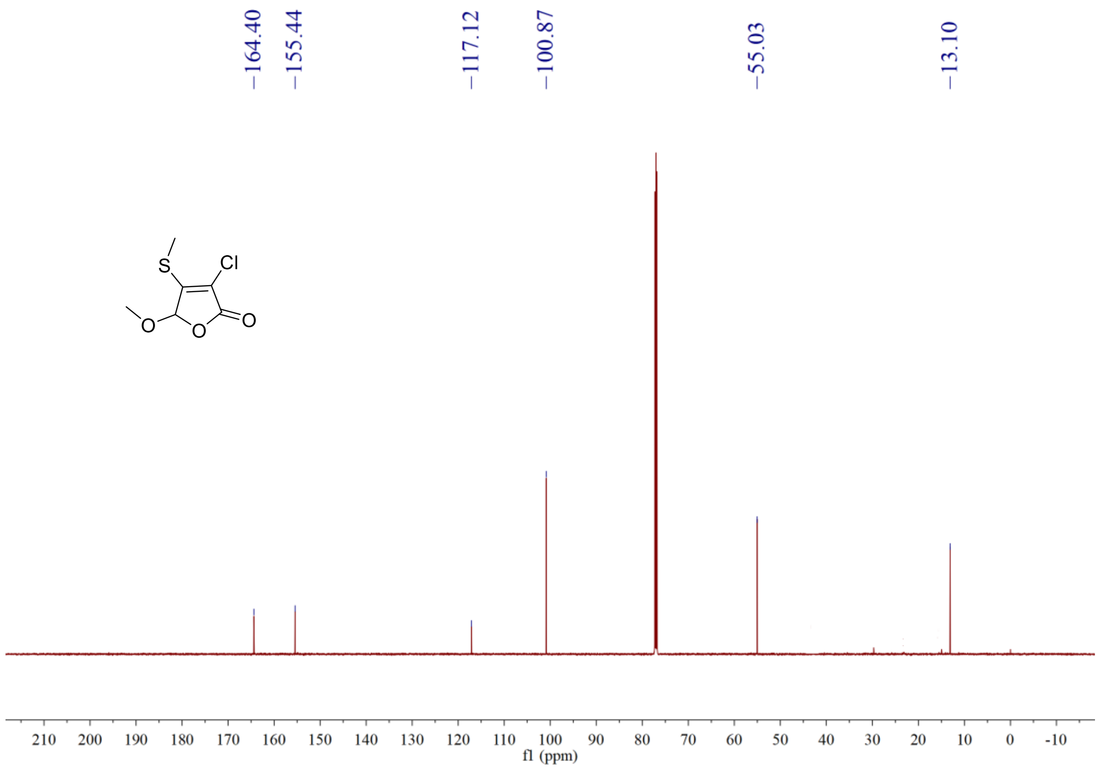

^{13}C NMR spectrum of compound **3l**

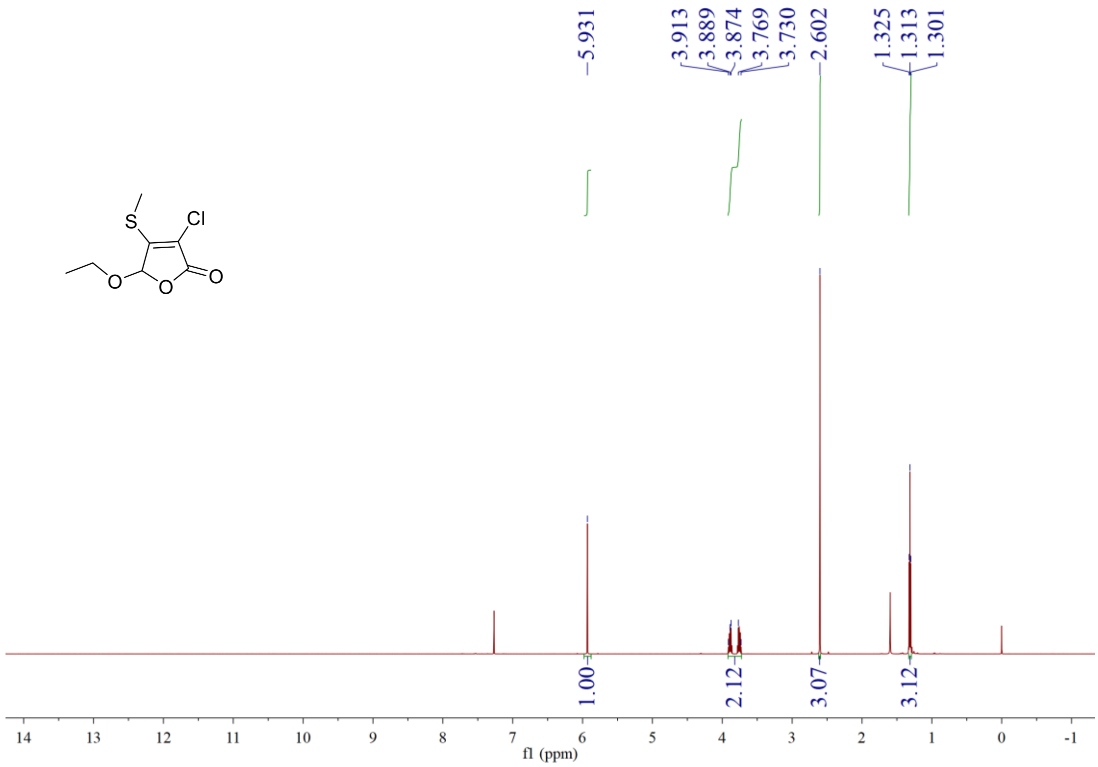

^{1}H NMR spectrum of compound **3m**

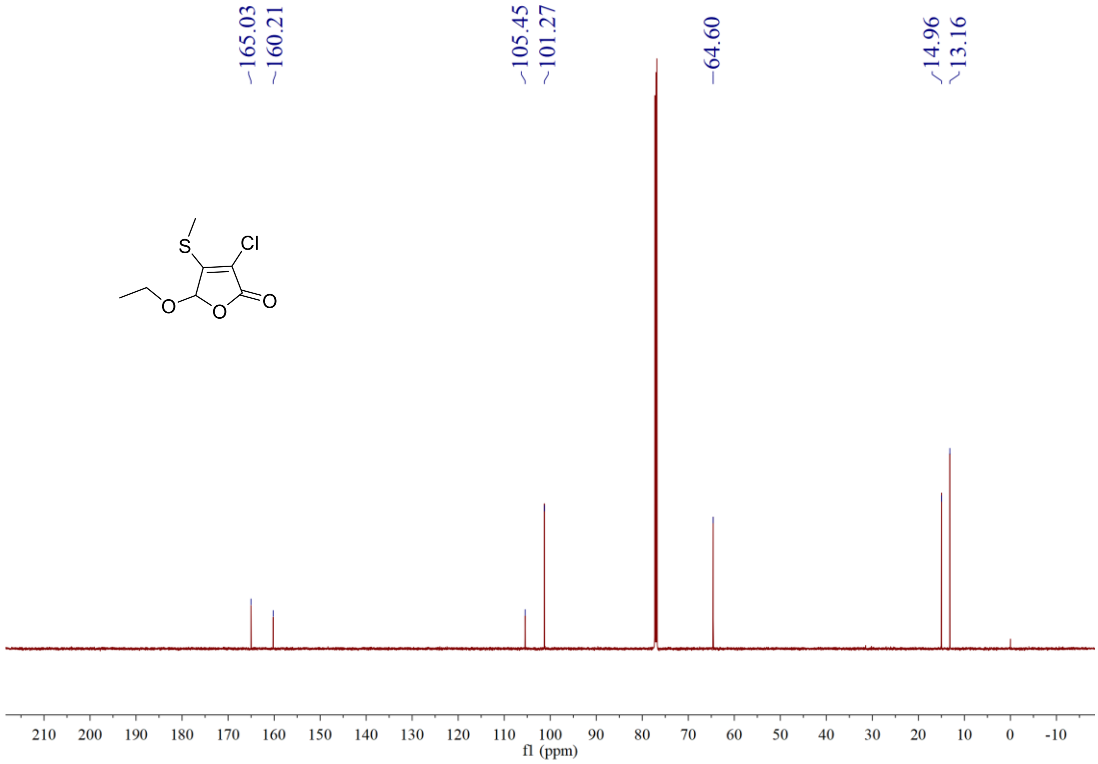

^{13}C NMR spectrum of compound **3m**

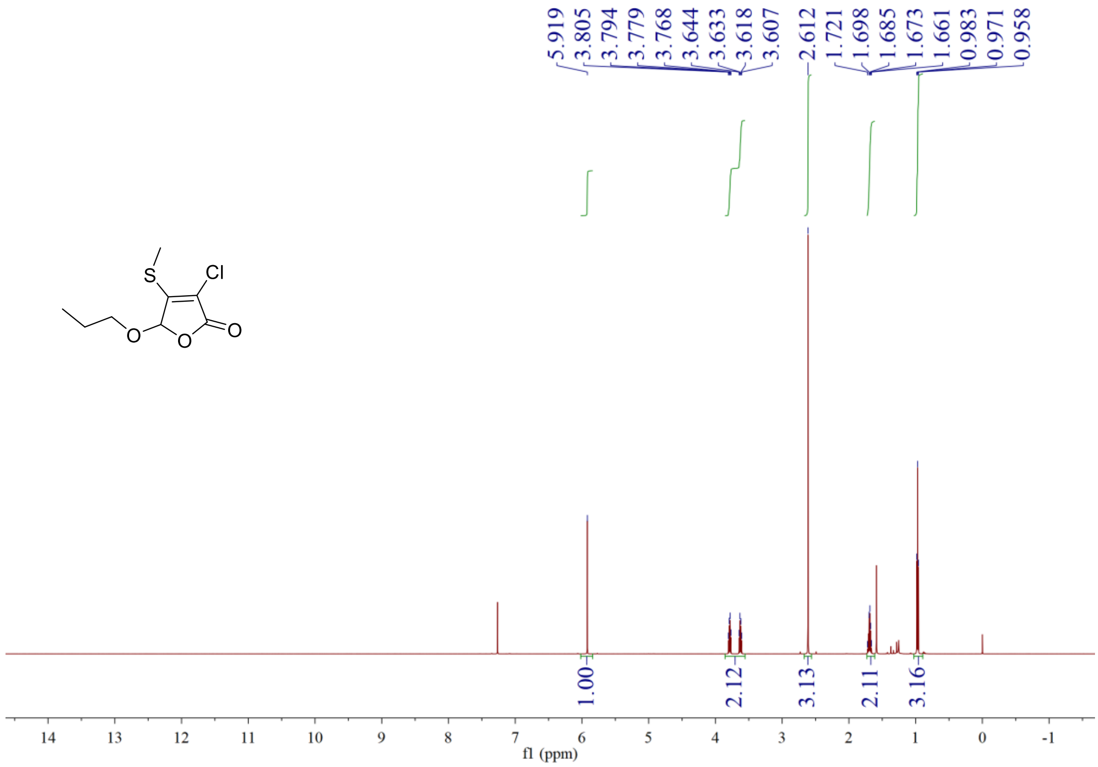

^{1}H NMR spectrum of compound **3n**

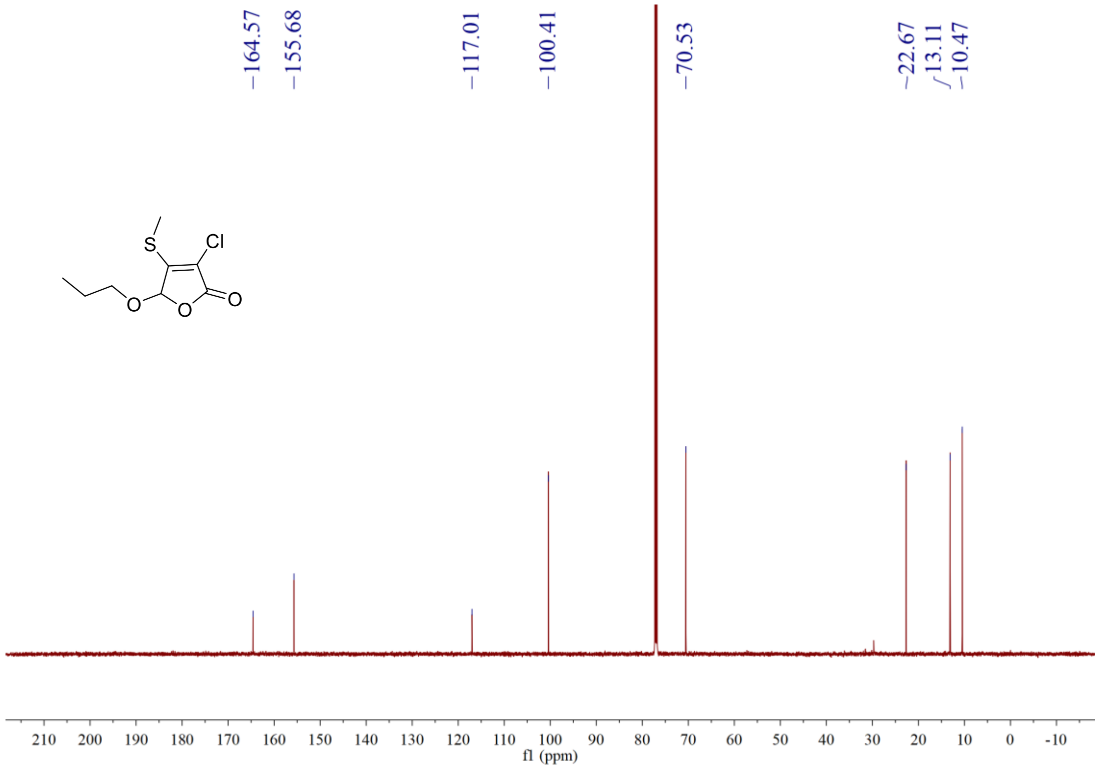

^{13}C NMR spectrum of compound **3n**

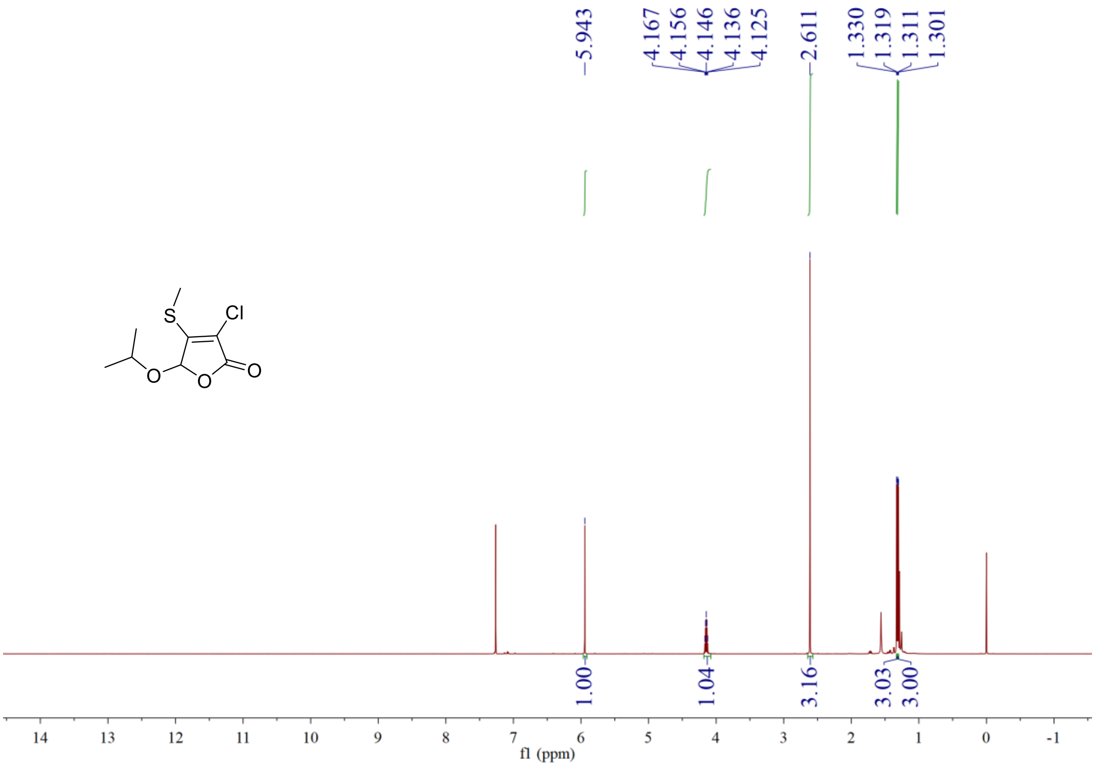

^{1}H NMR spectrum of compound **3o**

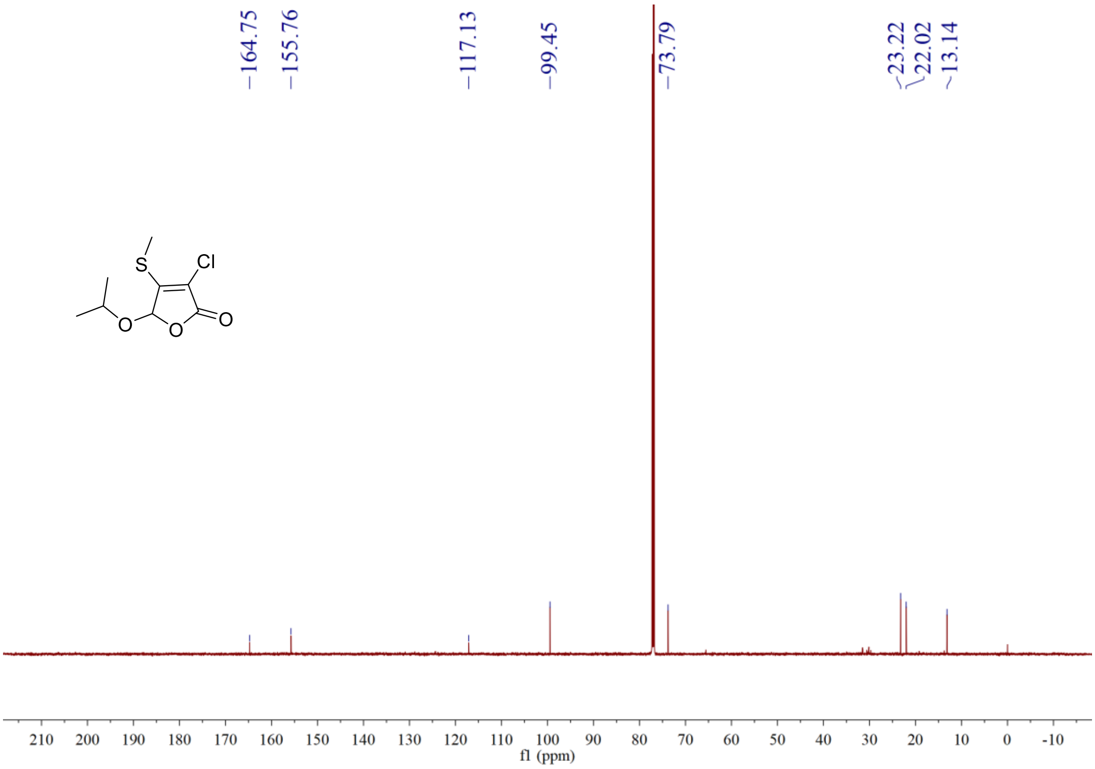

^{13}C NMR spectrum of compound **3o**

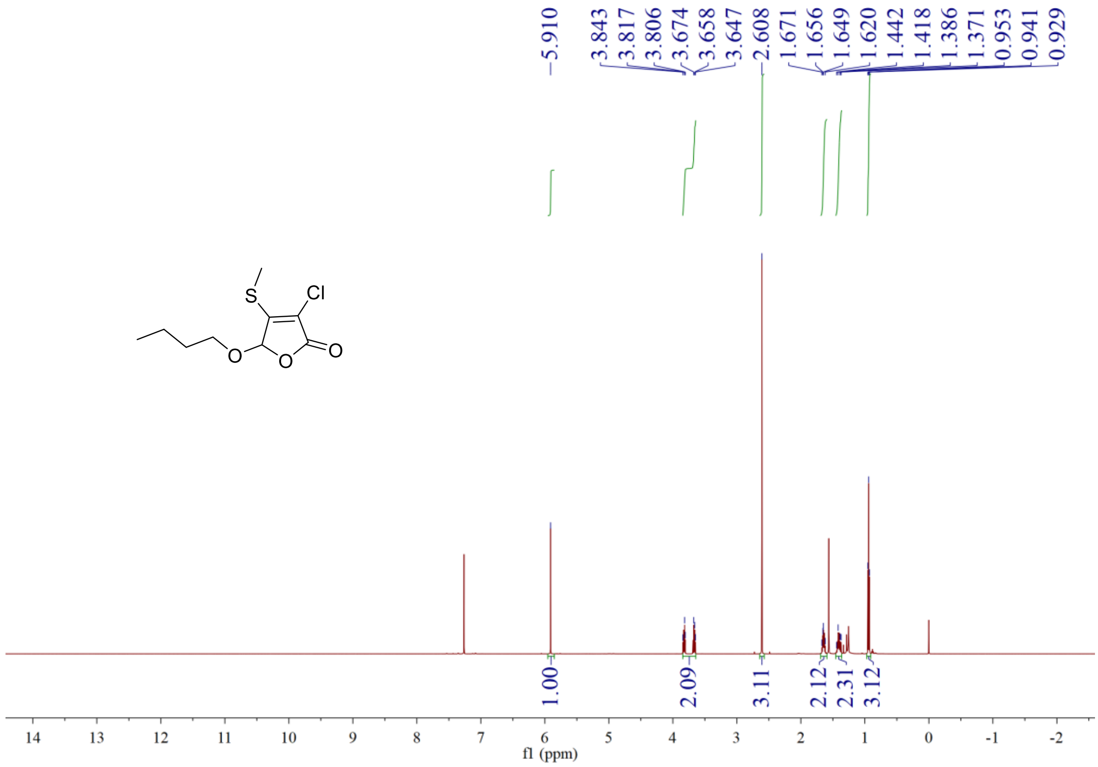

^{1}H NMR spectrum of compound **3p**

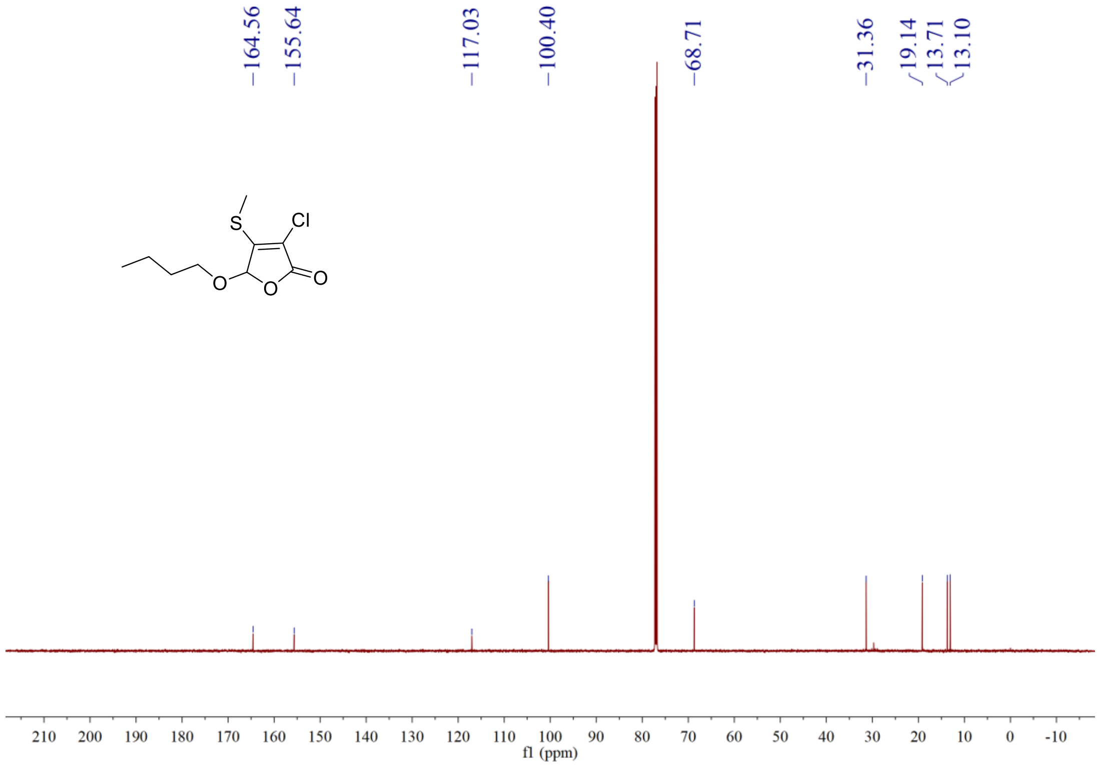

^{13}C NMR spectrum of compound **3p**

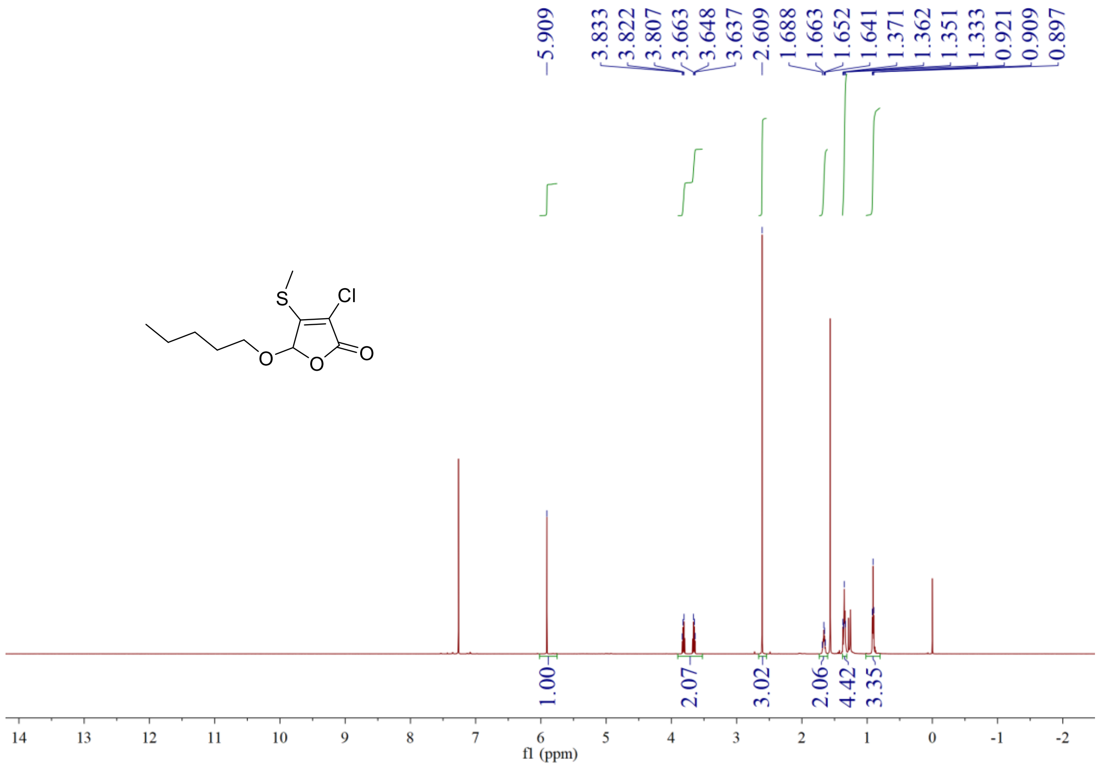

^{1}H NMR spectrum of compound **3q**

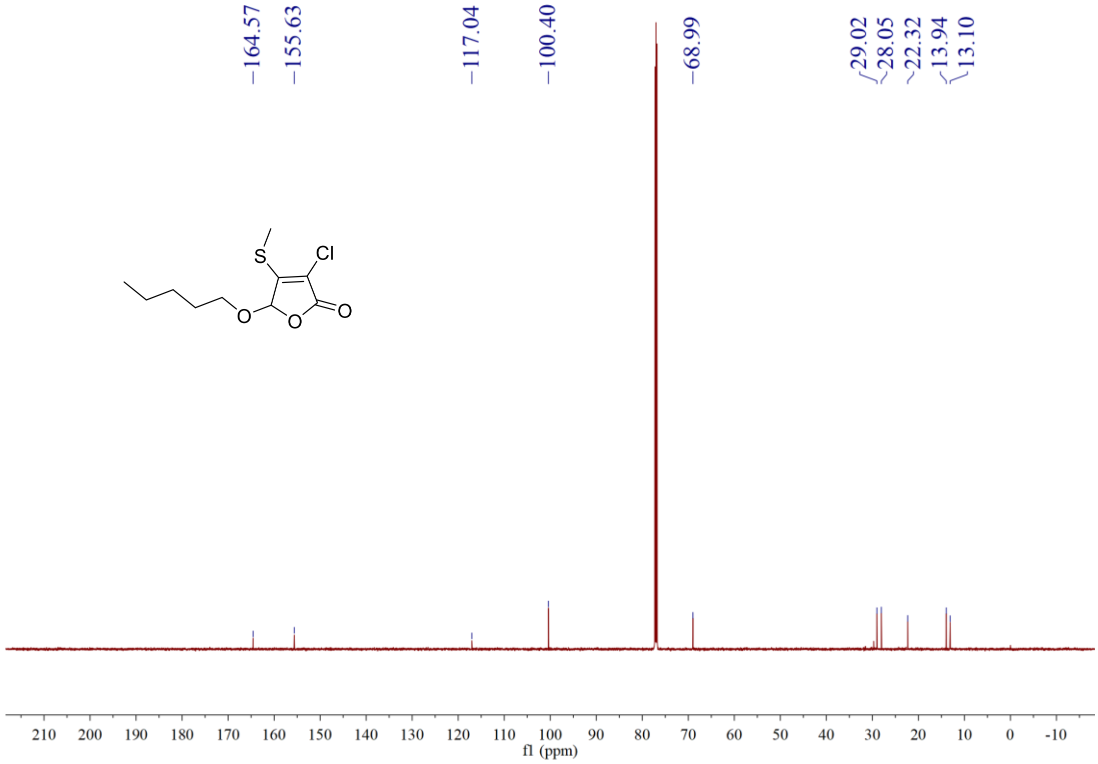

^{13}C NMR spectrum of compound **3q**

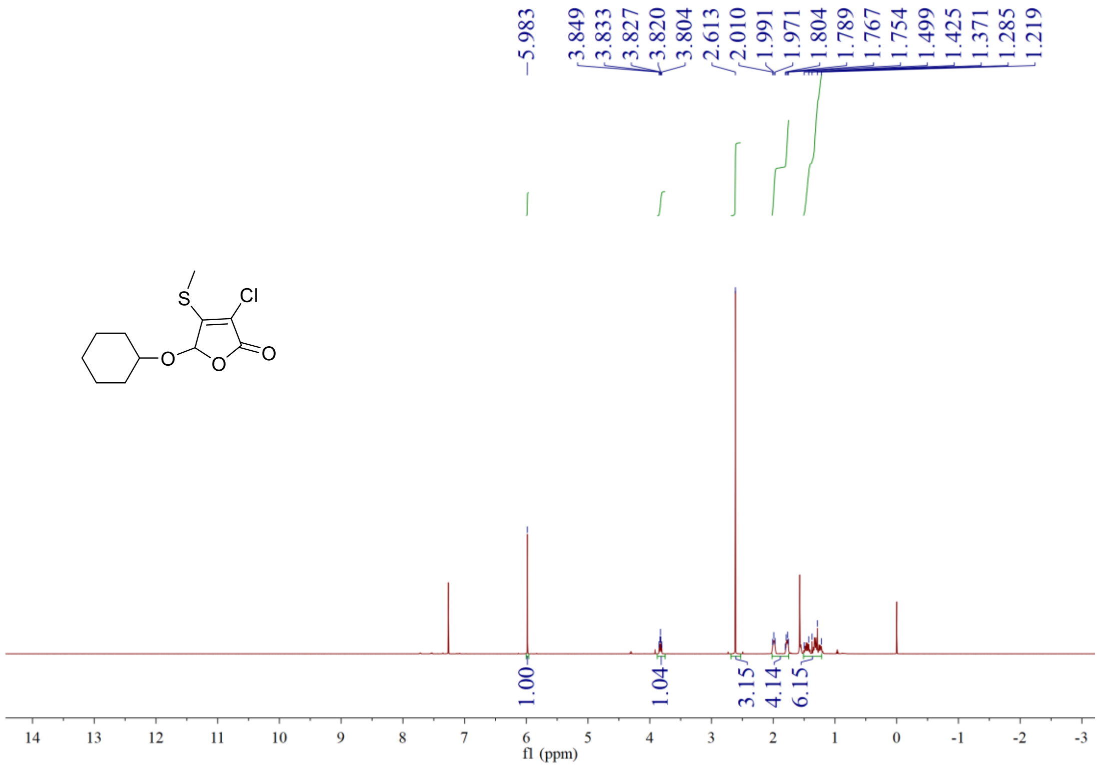

^{1}H NMR spectrum of compound **3r**

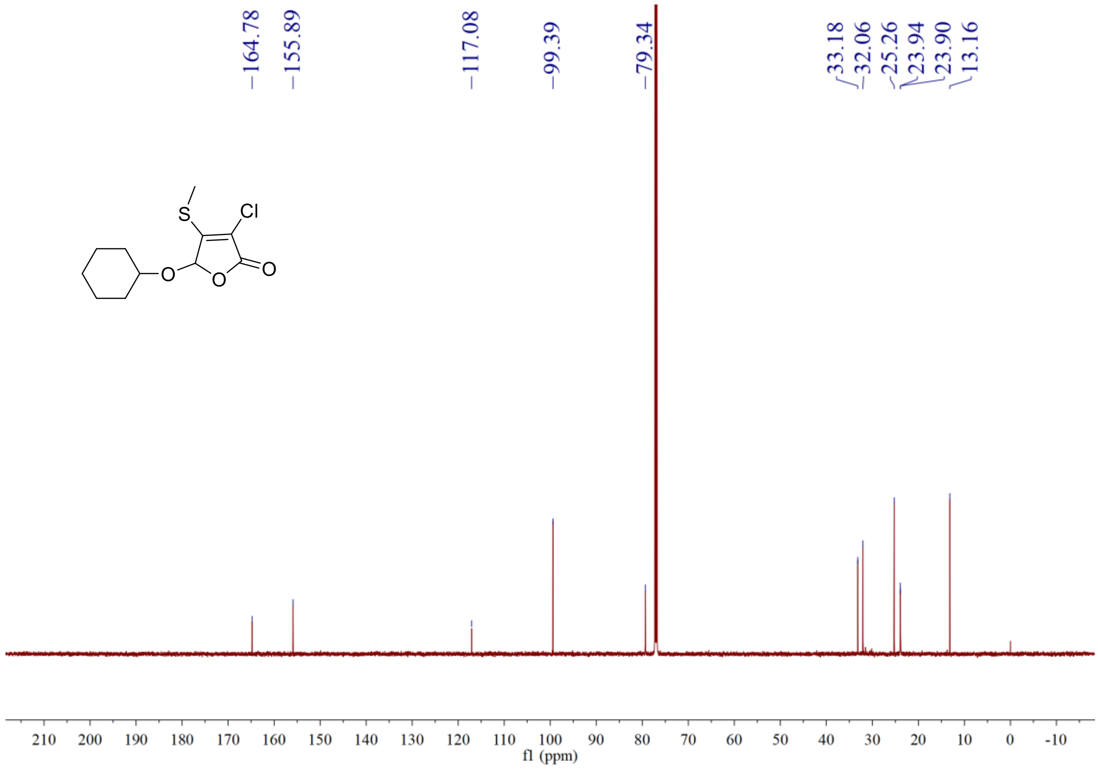

^{13}C NMR spectrum of compound **3r**

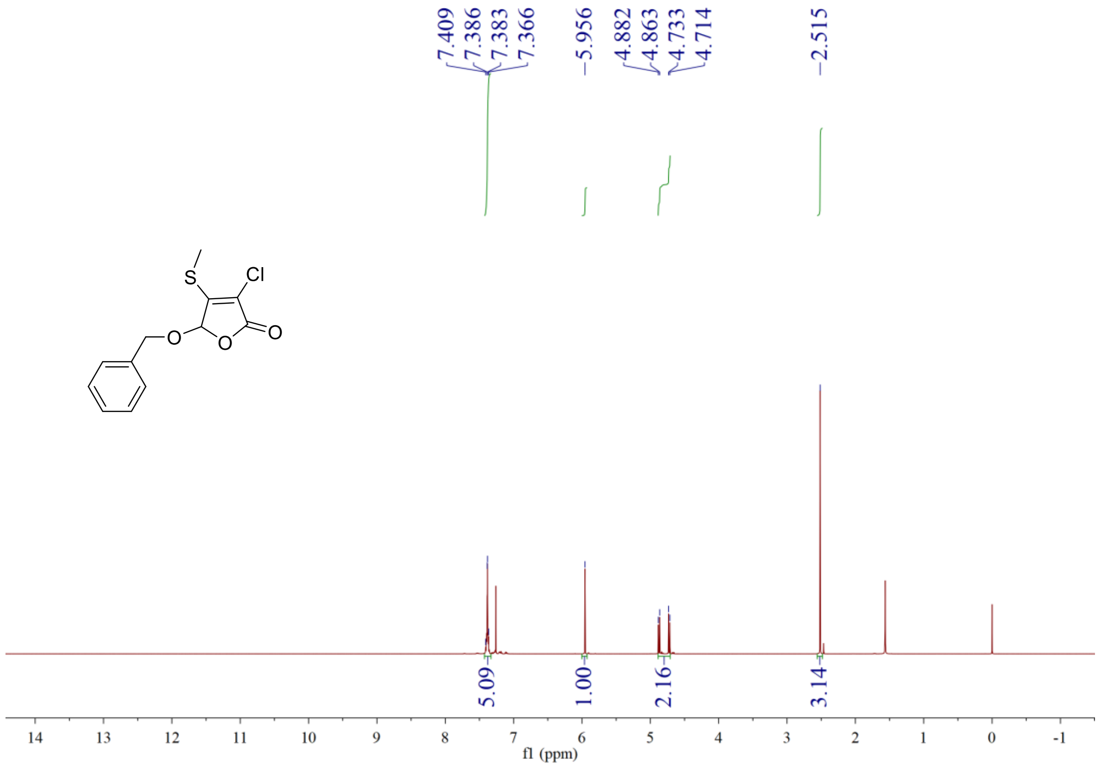

^{1}H NMR spectrum of compound **3s**

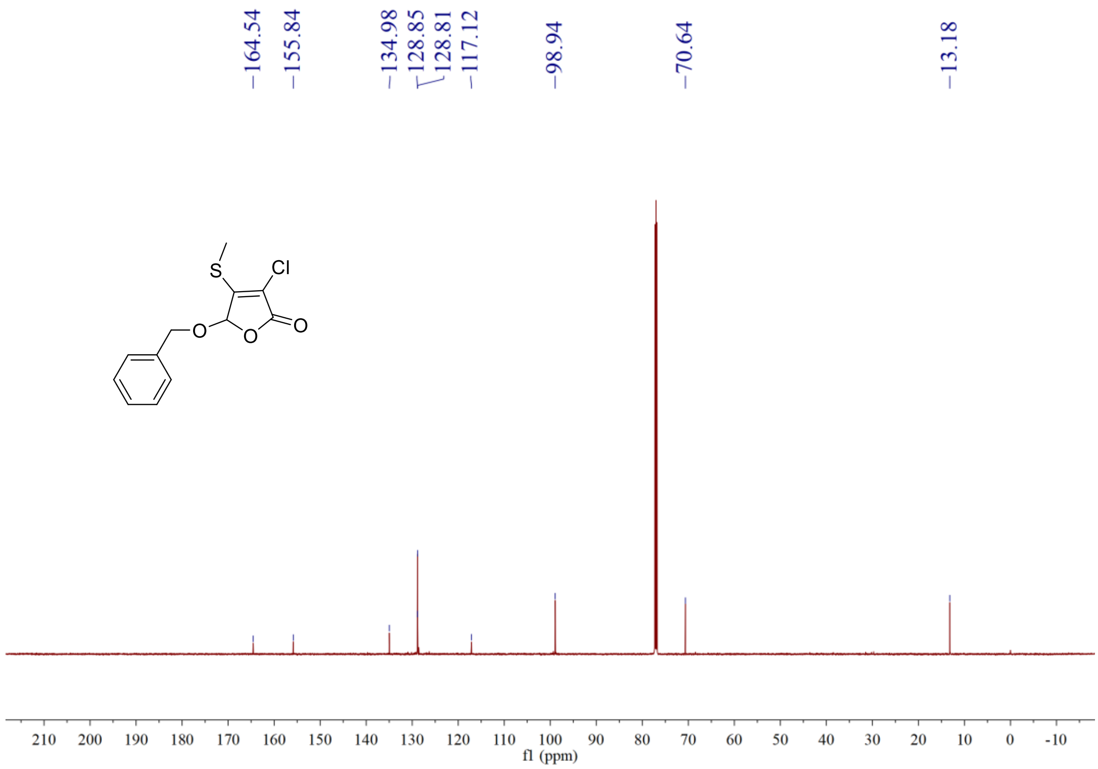

^{13}C NMR spectrum of compound **3s**

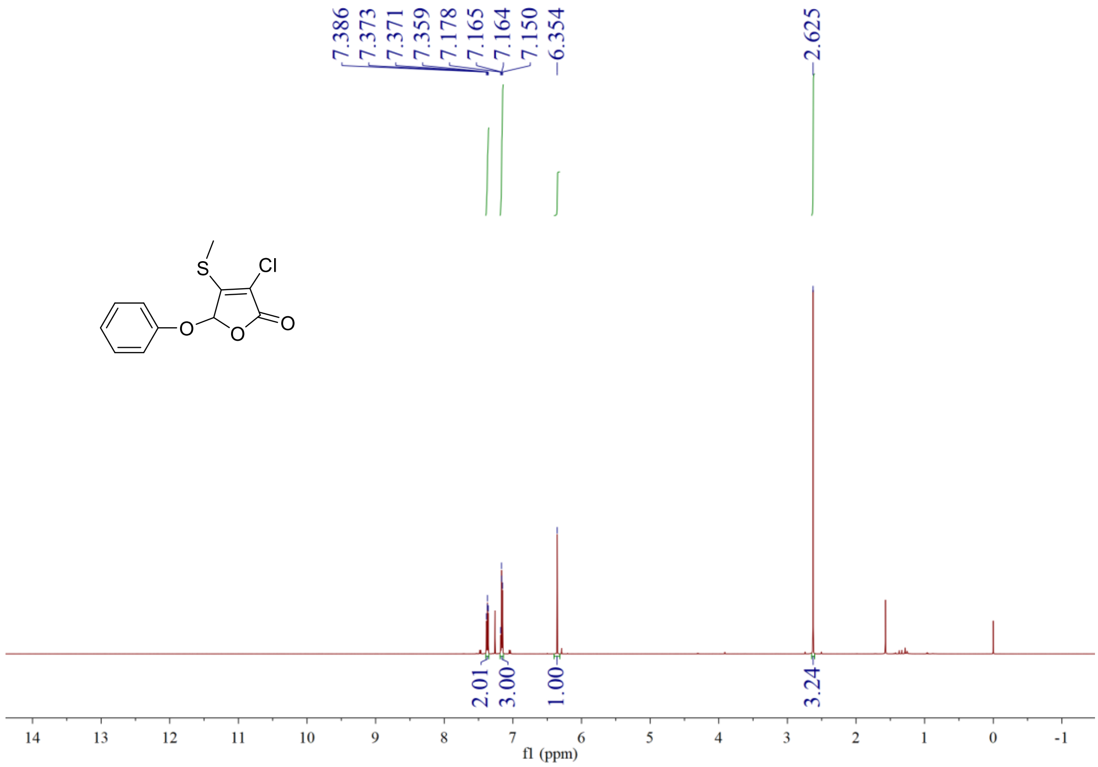

^{1}H NMR spectrum of compound **3t**

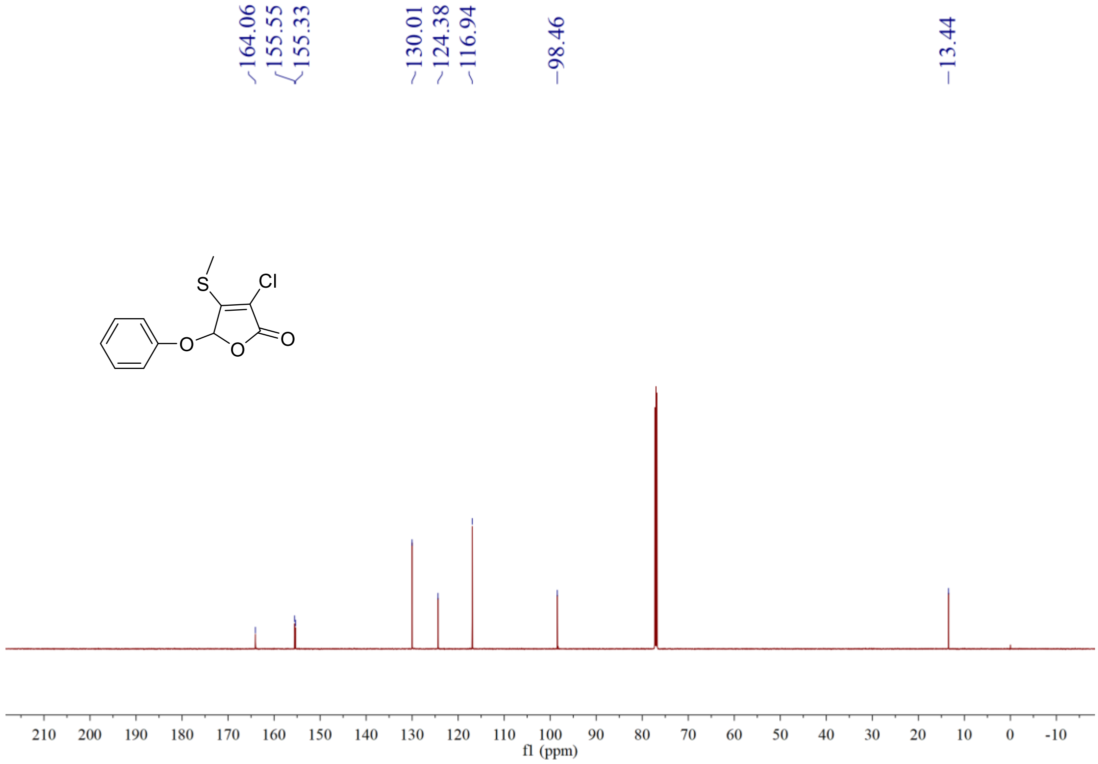

^{13}C NMR spectrum of compound **3t**
